# The basolateral amygdala-anterior cingulate cortex circuit contributes to postherpetic neuralgia-anxiety comorbidity

**DOI:** 10.7150/thno.111130

**Published:** 2025-03-21

**Authors:** Xiaofeng Jiang, Yi Yan, Ziming Chen, Jiaxin Xiong, Xuexue Zhang, Lili Gu, Yong Zhang, Mengye Zhu, Daying Zhang, Jian Jiang

**Affiliations:** 1 Jiangxi Key Laboratory of Trauma, Burn and Pain Medicine, Nanchang, China.; 2 Department of Pain, the First Affiliated Hospital, Nanchang University, Nanchang, China.; 3 Department of Radiology, the First Affiliated Hospital, Nanchang University, Nanchang, China.; 4 Neuroimaging Lab, Jiangxi Province Medical Imaging Research Institute, Nanchang, China.

**Keywords:** Postherpetic neuralgia, fMRI, Basolateral amygdala, Anterior cingulate cortex, Neural circuit

## Abstract

**Background:** Postherpetic neuralgia (PHN) causes chronic pain and emotional dysfunction, but its underlying mechanisms are unknown.

**Methods:** We first compared the structural and functional magnetic resonance imaging (MRI) of PHN-anxiety patients with healthy controls (HCs). Then, we created a PHN comorbid anxiety-like model by injecting resiniferatoxin (RTX) intraperitoneally and used Fos-CreER::Ai9 mice to validate brain regions with volume differences in MRI. Furthermore, we combined behavioral experiments with electrophysiology, viral tracing, *in vivo* fiber-photometry, optogenetics, and chemogenetics, to analyze the role of the basolateral amygdala (BLA)-anterior cingulate cortex (ACC) circuit in PHN comorbid anxiety-like mice multi-dimensionally.

**Results:** According to neuroimages, patients with PHN-anxiety comorbidity have decreased amygdala volume and decreased functional connection (FC) of the BLA and ACC. Furthermore, we created a PHN comorbid anxiety-like model by injection of RTX intraperitoneally, and these mice showed dysesthesia and anxiety-like behaviors 3 weeks after RTX injection. Then, we discovered that BLA and ACC were related to PHN comorbid anxiety-like behaviors using Fos-CreER::Ai9 mice. Immunohistochemistry and electrophysiology revealed enhanced activation of BLA glutamatergic (BLA^Glu^) neurons in PHN comorbid anxiety-like mice. Opto/chemogenetic activating BLA^Glu^ neurons aggravated pain threshold in PHN comorbid anxiety-like mice. Inhibiting BLA^Glu^ alleviates mechanical nociception, thermal hyperalgesia, and anxiety-like behavior. Moreover, the elevated excitability of BLA^Glu^ neurons resulted in increased excitatory inputs to the ACC. Selective activation or inhibition of the BLA^Glu^-ACC pathway exacerbated or alleviated the pain and anxiety behavior, respectively.

**Conclusion:** Findings in this study will provide new insight for understanding the central pathomechanism underlying PHN-anxiety comorbidity, as well as serve as solid theoretical underpinnings for the management of PHN.

## Introduction

Postherpetic neuralgia (PHN) is the most common and refractory sequela of herpes zoster, defined as pain lasting three months or more after the rash has healed 1, 2. PHN occurs in approximately 5 % - 20 % of herpes zoster patients 3 and is characterized by persistent severe pain, burning, or tingling 4. More than half of PHN patients have emotional disorders such as anxiety and depression, which can contribute to nociceptive hypersensitivity and impaired pain tolerance 5, 6 and make it difficult to treat. However, most previous investigations of pain and anxiety were conducted independently. The neurological mechanism underlying this comorbidity remains unknown.

Resting-state functional magnetic resonance (rs-fMRI) studies suggest that structural and functional brain abnormalities may be associated with pathophysiologic mechanisms of PHN-anxiety comorbidity 7, 8. In addition, previous studies have confirmed that the amygdala plays a vital role in pain perception and anxiety modulation 9, 10. However, the particular processes by which the amygdala participates in PHN-anxiety comorbidity remain unknown. The amygdala consists of structurally and functionally distinct subregions with separable connectivity features 11. According to cytoarchitectural maps of the human postmortem brain 12 and neuroimaging-based probabilistic map modeling 13, the amygdala can be classified into three main subregions, including the basolateral amygdala (BLA), centromedian amygdala (CMA), superficial amygdala (SFA). The BLA is the primary site for receiving and integrating multimodal information inputs from the cortex, thalamus, and other regions, which are then exported downstream to participate in regulation 14. It has been found that excitatory synaptic transmission of BLA is enhanced in rats with inflammatory pain, and inhibition of enhanced excitatory synaptic transmission rescued the pain threshold and alleviated pain-related aversive behaviors 15. In addition, anxiety behavior induced by chronic restraint stress is associated with dendritic hypertrophy and glutamate-related synaptic remodeling of BLA projection neurons 16. These studies indicate that excitability and synaptic transmission of BLA play a significant role in the development and maintenance of pain and anxiety.

The anterior cingulate cortex (ACC) is a critical area that is closely associated with the affective component of pain 17, which can be activated by nociceptive stimuli and shows abnormal activity in patients and animals with chronic pain 18, 19. Rs-fMRI studies have shown that the ACC is persistently activated during painful states and correlates with the degree of pain 20. Activation of ACC neurons has been associated with mood disorders 21 or pain-like aversive 22 behavior. Studies in rodents have shown that ACC neurons are activated by sensory stimuli and that presynaptic long-term potentiation (LTP) is linked to pain-induced anxiety 23, 24. Optogenetic activation of pyramidal neurons in ACC is sufficient to induce anxiety-like behavior in naive mice 21. Inhibition of ACC plasticity produces analgesic and anxiolytic effects in different animal models of chronic pain and anxiety 18. Thus, the ACC may be a key brain region for chronic pain and anxiety behaviors. BLA and ACC are associated with pain and mood disorders, and the BLA-ACC circuit is involved in behaviors such as pain 25, anxiety 26, and depression 27, but it is unclear whether the BLA-ACC circuit contributes to the PHN-anxiety comorbidity.

Based on the above research, we first compared the neuroimaging data of PHN-anxiety comorbidity patients with healthy controls (HCs) and found that the structure of the amygdala was altered in PHN-anxiety patients. We further divided the amygdala into three subregions as seed points to do the functional connectivity (FC) with the whole brain, which revealed that the decreased FC of the BLA with ACC, and the FC value was correlated with the Visual Analog Scale (VAS) 28 and Hamilton Anxiety Scale (HAMA) 29. Moreover, we created a PHN comorbid anxiety-like model by injecting resiniferatoxin (RTX) intraperitoneally to mimic the pain and anxiety behavioral phenotypes associated with PHN 30, and these mice developed dysesthesia and anxiety-like behaviors after 21 days. Then, we utilized Fos-CreER::Ai9 mice to validate brain regions with volume differences in fMRI. Furthermore, we combined behavioral experiments with electrophysiology, viral tracing, in vivo fiber-photometry, optogenetics, and chemogenetics to analyze the role of the BLA-ACC circuit in PHN comorbid anxiety-like mice multi-dimensionally. This study has the potential to expand the knowledge and understanding of the mechanism of PHN-anxiety comorbidity, as well as ameliorate the clinical dilemma of PHN.

## Materials and Methods

### Magnetic resonance experimental methods

1) Subjects and grouping: PHN-anxiety comorbidity patients were recruited from the pain department of the First Affiliated Hospital of Nanchang University between March 2020 and December 2023. The diagnosis of PHN was based on the criteria of the International Association for the Study of Pain (IASP)^31^. The inclusion criteria were as follows: (1) confirmed diagnosis of PHN (skin lesion recovery and pain duration more than 3 months); (2) all patients had a VAS ≥ 5 and a HAMA score ≥ 14; (3) No antidepressants or antipsychotics were taken before the MRI scan; (4) All patients were treated with first-line drugs for PHN (pregabalin and/or tramadol) only before MRI scanning. Exclusion criteria were as follows: (1) other chronic pain or neurological disorders; (2) suffering from neurological or psychiatric disorders; (3) history of head injury, alcohol or drug abuse; and (4) contraindications to MRI scanning. Age-, gender-, and education-matched HCs were also recruited, and HCs did not have any spontaneous pain or sensory abnormalities, neurologic or psychiatric disorders, history of substance abuse, or structural brain abnormalities. All subjects were right-handed. Subjects were excluded due to excessive head motion or partial image loss. Clinical data of the participants were collected before the MRI scan, and the intensity of the patient's spontaneous pain was assessed using the VAS, and the HAMA was used to assess the state of anxiety in all participants. This study strictly adhered to the Declaration of Helsinki and was approved by the Ethics Committee of the First Affiliated Hospital of Nanchang University (20200145).

2) Voxel-based morphometry (VBM): VBM analysis was performed using the CAT12 toolkit (https://neuro-jena.github.io/cat) based on SPM12 (http://www.fl.ion.ucl.ac.uk/spm). Before processing, the dcm2nii toolkit was used for data format conversion to convert DICOM images to NIFIT. The specific processing flow of VBM includes the following steps: (1) tissue segmentation, using the DARTEL algorithm to segment the brain into gray matter (GM), white matter, and cerebrospinal fluid; (2) spatial normalization, aligning the segmented GM images to the Montreal Neurological Institute (MNI). (3) spatial standardization, the segmented GM images were aligned to the MNI standard space to facilitate group-level statistical analysis; (4) modulation, to correct the bias generated in the spatial standardization process; (5) after the completion of the preprocessing pipeline, quality checking was carried out using the CAT12 toolbox to assess the homogeneity of the GM tissues. (6) Smoothing: the corrected images were smoothed using an 8 mm smoothing kernel to analyze GM volume differences further.

3) FMRI data preprocessing: image preprocessing was performed on the Maltlab 2018b (Math Work, Natick, MA, USA) platform using the SPM12 toolkit. The specific process included (1) DICOM format conversion; (2) exclusion of the first 10 time points; (3) interlayer time correction; (4) head movement correction; (5) alignment of the high-resolution T1 structural images to the standard Montreal space, separation of the brain white matter, brain gray matter, and cerebrospinal fluid on T1 structural images using the DARTEL segmentation algorithm, and one by one, alignment of the tissue T1 images to the functional images, and resampling for 3 mm isotropic voxels; (6) de-linearization drift; (7) removal of covariates (24 cephalometric parameters, cerebral white matter, and cerebrospinal fluid signals, and whole-brain signals) using linear regression; (8) final use of filtering (0.01 to 0.08 Hz) to reduce low-frequency drift and physiological noise at high frequencies; and (9) spatial smoothing using half-peak full-width (FWHM) = 6 mm to translate cephalic motion by > 2 mm and/or rotation > 2° were excluded from the subject data for subsequent analysis.

4) FC analysis: the amygdala subregion was extracted as a seed point in the Julich histological atlas 12, ROI-wise FC was calculated using the SPM12-based DPABI (http://rfmri.org/dpabi) toolkit, and FC was quantified using the Pearson's correlation coefficient (r) to indicate the correlation between the mean time series of each ROI and the mean time series of other voxels. All r-values were converted to Z-values using the Fisher'Z transformation to improve the normality.

### Animals

For targeted recombination in active populations, second-generation male Fos-CreER::Ai9 mice aged 6 to 8 weeks were employed in this experiment. The Fos-CreER mice (Geneandpeace, Jiangsu, China) were crossed with Ai9-TdTomato Cre-reporter mice (Geneandpeace, Jiangsu, China) to visualize active neurons. Adult male C57BL/6 mice (age 6 - 8 weeks) weighing 20 - 25 g were used in other experiments. The C57BL/6 mice were acquired from Jiangsu Jicui Pharmachem Biotechnology Co. All mice were housed according to a 12 h light/12 h dark cycle (lights on at 8:00), with the room temperature controlled at 22 - 25 ℃, and food and water were freely available. Every procedure complied with the National Institutes of Health's Guide for Care and Use of Laboratory Animals as well as the Committee for Research and Ethical Issues of IASP.

### Animal models of PHN

The PHN-like model was established via a single intraperitoneal injection of RTX at a dose of 25 ug/kg, which was dissolved in a mixture of 10 % Tween80, 10 % ethanol, and saline 30. The same dose of solvent solution was injected into controls. The mechanical and thermal pain thresholds of each mouse were measured before and 21 days after RTX and solvent injection. RTX was purchased from CFW LABS in the United States.

### Pain and anxiety behavioral testing

#### 1) Von-Frey test

Before testing, mice were placed inside a Plexiglas cage (10 cm × 10 cm × 13 cm) with a metallic mesh floor and allowed to acclimate for 30 minutes. A series of calibrated Von Frey filaments (0.02 - 2.56 g, North Coast Stoelting, USA) was applied to the right hindpaws in increasing order, and the 50% paw withdrawal threshold (PWT) was determined 32. Mice demonstrating quick foot retraction, lifting, or foot licking on the hind foot were regarded as positive responses. The next stronger filament was applied if the chosen test filament did not produce a positive reaction. If the paw retreated, the following weaker stimulus was chosen.

#### 2) Hargreaves test

Mice were placed in a transparent test box for at least 1 hour to accommodate the testing environment. Heat stimulation was applied at infrared intensity (IR) 32 with a 30-second cut-off time. The paw withdrawal latency (PWL) was calculated as the time between the initiation of the beam light and the animal withdrawal of the paw from the heat stimulus. The stimulation was performed three times, with a 5-minute interval between each, and the average value was taken.

#### 3) Open field test (OFT)

The test was conducted in an uncovered square box of 50 cm × 50 cm × 25 cm. All experimental mice were acclimatized in the behavioral test room for 1 hour before the test, while during the test, they were placed in the center of the box. The activity of the animals in the box was recorded for a 10-minute trial period. At the end of the test for each mouse, the box was thoroughly cleaned with 75% alcohol, and the odor and excreta were evaporated entirely before proceeding to the next mouse. The 36% area in the center of the box wall was designated as the central area. The video was analyzed using VisuTrack to record the total distance and the central time spent.

4) Elevated plus maze test (EPM)

The elevated cross maze is composed of two open arms and two closed arms. The width and the length of the arm are 5 cm and 30 cm respectively, and the height of the closed arms is 15 cm. The experiment lasted 5 minutes and VisuTrack was applied to track the animals' movement trace in the elevated plus maze offline. The time that the mice stayed in the open arms and the entries they entered the open arms were analyzed.

5) Light and dark box test (LDB)

The light and dark box (42 cm × 21 cm × 25 cm) were divided into two equal-size compartments: a light box and a dark box; the partition wall between the two boxes had a 5.0 cm×3.0 cm doorway for the animals to pass through. The mice were placed in the center of the dark box at the beginning, and the number of times the mice entered the light box, the retention time in the light box, and the latency to the light box were recorded within 5 min.

### Activity-dependent neuron labeling

Fos-CreER::Ai9 mice were used to label activated neurons in PHN comorbid anxiety-like mice. 4-hydroxytamoxifen (4-OHT) was injected intraperitoneally (50 mg/kg) 1 h before the EPM and LDB behavioral tests, respectively, and the mice were left in quiet and familiar surroundings for at least 6 h after the event. Mice were sacrificed the day after LDB. 4-OHT (H6278 Sigma USA) was prepared in castor and sunflower oil solutions as described in previous research 33.

### Immunohistochemistry (IHC)

Mice were anesthetized with urethane (1.5 g/kg, i.p.). Cardiac perfusion was performed successively with saline and 4 % PFA. After perfusion, the brains were removed and post-fixed in 4 % PFA overnight and dehydrated in 30 % sucrose for 1 week. Mouse brains were serially cut into 40-μm thick coronal slices by a frozen microtome (Leica CM 1950, Germany). Brain slices were washed three times in phosphate-buffered saline (PBS) for 10 min each time and then incubated in a buffer containing 0.3 % Triton X-100 (X-100, Sigma, USA), 1 % bovine serum albumin (A4503, Sigma, USA), and 1 % normal donkey serum (ab7475, Sigma, USA) for 1 h at room temperature. The sections were incubated with primary antibodies, including anti-c-FOS (2250S, 1:1000, Rabbit, Cell Signaling Technology, USA or 226308, 1:5000, guinea pig, SYSY, Germany), anti-Glutamate (G6642, 1:500, Rabbit, Sigma-Aldrich, USA) for 3 days at 4 °C. Then the sections were washed with PBS (10 min×3 times), and the secondary antibodies of the corresponding species were added for incubation at room temperature for 1 h, including Cy5 (706-175-148, 1:400, guinea pig, Jackson ImmunoResearch, USA), Alexa Fluor 647 (A31573, 1: 400, Rabbit, Invitrogen, USA). Finally, the samples were washed with PBS (10 min×3 times). The brain sections were mounted and counterstained with DAPI (0100-20, Southern Biotech, USA). The images were acquired and processed using ZEN software (Zeiss) on an LSM700 confocal microscope (Zeiss) and further analyzed by Image J software. All data points for statistical analysis were derived from an individual slice of independent mice.

### Virus injection

Under isoflurane anesthesia, mice were placed on a stereotaxic frame (RWD Life Science). The injection coordinates were selected according to the Atlas of the Mouse Brain. The stereotaxic coordinates were defined as dorsoventral (DV), anteroposterior (AP), and mediolateral (ML) coordinates (in millimeters). The brain regions and coordinates (AP, ML, DV) of the injection targets were as follows: BLA: (- 1.30, ± 3.20, - 4.3), ACC: (+ 0.75, ± 0.30, - 1.40).

### Preparation of brain slices

Mice were anesthetized, and cardiac perfusion was performed with ice-cold sucrose-based artificial cerebrospinal fluid (s-ACSF) (mM: 75 sucrose, 25 NaHCO_3_, 80 NaCl, 2.5 KCl, 1.25 NaH_2_PO_4_·2H_2_O, 0.5 CaCl_2_·2H_2_O, 3.5 MgCl_2_·6H_2_O, 0.4 ascorbic acid, 2 pyruvate) saturated with oxygen. After perfusion, the brain was rapidly removed and wholly immersed in s-ACSF, and coronal target brain slices with a thickness of 280 μm were cut by a vibrating microtome (VT1000S, Leica, Germany) at a speed of 0.16 mm/s. Then, the brain slices were transferred to oxygenated ACSF (mM: 117 NaCl, 3.6 KCl, 1.2 NaH_2_PO_4_·2H_2_O, 2.5 CaCl_2_·2H_2_O, 1.2 MgCl_2_·6H_2_O, 25 NaHCO_3_, 11 glucose, 0.4 ascorbic acid, 2 pyruvate, pH = 7.4) at 32 ℃ to incubate for 30 minutes, followed by incubation for 30 min at room temperature.

### *In vitro* electrophysiological recordings

Brain slices were placed in a recording chamber and continuously perfused with oxygenated ACSF at a rate of 2.5 - 5 mL/min. Patch pipettes were pulled from borosilicate glass capillaries (1.5 mm OD, 1.12 mm ID, World Precision Instruments, USA) by Micropipette Puller (MP-500, RWD Life Science). Whole-cell recordings were made with K^+^-based intracellular solution, which consisted of (mM: 130 K-gluconate, 5 KCl, 0.5 EGTA, 10 HEPES, 4 Mg-ATP, 0.3 Li-GTP, 10 phosphocreatine, pH = 7.2), and the resistance of the recording electrodes was 5 - 8 MΩ after filling the electrode solution. Patchmaster software (HEKA Electronics, Lambrecht, Germany) and an EPC-10 amplifier were used to conduct patch-clamp recordings. Neuronal active and passive membrane properties, action potentials (AP), input resistance (Rin), and excitatory postsynaptic currents (EPSC) were recorded. Data were analyzed using Clampfit 10.7 (Molecular Devices, USA).

To analyze the electrophysiological properties of BLA^Glu^ neurons projecting to ACC (BLA^ACC^) neurons, AAV2/Retro-CaMKIIα-mCherry was injected into the ACC region, and mCherry-infected BLA^ACC^ neurons were identified by a 590 nm LED (M590L4, Thorlabs, USA) light source.

### *In vitro* optogenetic electrophysiology

Optical stimuli were emitted by LEDs connected to a stimulator (Master 8) controlled by the Pacthmaster software and projected onto the surface of the recording brain slices. To verify the function of AAV-CaMKIIα-ChR2-mCherry, mCherry+ neurons in the targeted brain region were activated by blue laser stimulation (470 nm, 10 uW, 1 ms). A yellow laser (590 nm, 10 uW, 500 ms) was employed to verify the function of AAV-CaMKIIα-eNPHR-mCherry. To validate monosynaptic connections, the AAV-CaMKIIα-ChR2-mCherry virus was injected into the BLA, and electrophysiological recordings were made from neurons in the ACC. The optogenetic excitatory postsynaptic currents (oEPSC) were recorded in voltage-clamp mode (-70 mV). Sodium channels were blocked by perfusion of 1 μM tetrodotoxin (TTX), followed by additional application of 1 mM 4-aminopyridine (4-AP). The oEPSC was confirmed as an excitatory postsynaptic current by applying the AMPA receptor antagonist CNQX (100 μM). For presynaptic transmission analysis, two consecutive blue laser stimuli (470 nm, 10 μM, 1 ms), spaced 100 ms apart, were given to assess the paired pause ratio (PPR). After the experiment, brain slices were examined by fluorescence microscope to confirm the injection location.

### *In vitro* chemogenetic electrophysiology

To verify the function of AAV-CaMKIIα-hM3dq/hM4di-mCherry, the virus was injected into the BLA, and recordings were made in the current clamp mode (0 pA). Brain slices were perfused with Clozapine N-oxide (CNO; Tocris, Bristol, UK), and spontaneous spikes were recorded. The CNO was dissolved to a concentration of 10 μM in ACSF.

### *In vivo* fiber-photometry

AAV-CaMkIIα-GCaMp6s-WPRE viruses were injected into the ACC with fiber optics implanted. AAV2/Retro-CaMkIIα-Cre-WPRE viruses were injected into ACC, AAV2/9-hsyn-DIO-GCaMp6s-WPREs viruses were injected into BLA, and fiber optics were implanted in BLA. Then the Ca^2+^ activity in ACC or BLA was recorded *in vivo* using a Multi-Channel Fiber Optic Recording System (R811/821, RWD Life Science) 3 weeks after injecting the fluorescent Ca^2+^ indicator GCaMP6s. The recording wires were installed the day before the test to allow the mice to acclimate to the recording procedure. To record the neuronal activity in response to mechanical stimulation, mice were stimulated with 0.6 g von Frey filament in the hind paw. For assessing neuronal activity during EPM, transitions from the closed arm to the open arm were defined as positive events. Calcium-dependent fluorescence signals from GCaMP6s were recorded using a 470 nm laser. The state data of average Ca^2+^ transient were acquired during pre-event (-2 - 0 s) and post-event (0 - 6 s). The Z-values of fluorescence changes (ΔF/F) and the area under the curve of the transitory Ca^2+^ change corresponding to the event were analyzed, with heat maps and average Ca^2+^ trajectories plotted.

### *In vivo* optogenetic/chemogenetic manipulation

1) To examine the immediate effects of modulating the BLA^Glu^ somata and BLA-ACC fibers on pain and anxiety-like behavior in PHN comorbid anxiety-like mice, opto/chemogenetic viruses or control viruses were injected into the BLA. For *in vivo* optogenetic manipulation, fiber optics were implanted in the BLA or ACC bilaterally to modulate somata or fibers, respectively. The coordinates for fiber optic implantation in the ACC were as follows: AP: 0.75 mm, ML: 1.0 mm, DV: 1.6 mm (at a 30° angle). For *in vivo* chemogenetic manipulation, pain and anxiety behaviors were tested after an intraperitoneal injection of CNO (3 mg/kg), aimed at modulating BLA somata. To modulate the BLA-ACC fibers, a dual-guide cannula (plastic cannula) was implanted into the ACC with a 0.6 mm side-by-side distance (RWD Life Science). The cannula was fixed with dental cement. To prevent blockages during the recovery period, a dummy cannula, 0.5 mm longer than the guide cannula, was inserted into the guide cannula and sealed with dust caps. A Hamilton micro-syringe injected CNO (10 μM) into the bilateral ACC. The injection needle was inserted into the guide cannula, and 200 nl CNO was infused at 100 nl/min. The injector cannula was left in place for 10 min to allow proper diffusion of CNO. Behavioral tests were performed 30 min after injection.

2) To precisely modulate BLA^ACC^ neurons, retrograde viruses were first injected into the ACC, followed by the injection of cre-dependent opto/chemogenetic viruses or control viruses into the BLA. Optical fibers were implanted into the BLA using the optogenetic method. In the chemogenetic method, pain and anxiety behaviors were assessed 30 min after intraperitoneal injection of CNO (3 mg/kg). Optogenetic equipment and settings were used in our previous study 34. Ferrules were connected (via patch cords) to the optogenetic bright light source (Aurora 300, NEWDOON INC, China) through an FC/PC adaptor and a fiber optic rotary joint. A 470 nm (20 mW, 15 ms pulses, 20 Hz) blue laser was used for activation, while a 589 nm (20 mW, 15 ms pulses, 20 Hz) yellow laser was used for inhibition. To ensure the reliability of the results, the brains were sectioned post-experiment to confirm the locations of the virus, optical fibers, and cannulas. Data were discarded if the positions deviated from the targeted brain regions.

### Statistical analysis

Statistical analysis was performed using GraphPad Prism (version 9.0, GraphPad Software, San Diego, CA, USA). Data were tested for normality and homogeneity using the Shapiro-Wilk and Levene's tests. Continuous variables from the clinic that obey normal distribution were expressed by mean ± SD; otherwise, median-interquartile spacing was used. The chi-square test was used for the categorical variable. VBM: Using age, gender, and total intracranial volume as covariates, a two-sample t-test was conducted in SPM 12 to compare the gray matter volume between PHN-anxiety patients and HCs. The multiple comparisons correction was performed using the Family-Wise Error correction, with a significance threshold set at *p* < 0.05. FC: Using age, gender, and education level as covariates, a two-sample t-test was performed with Gaussian Random Field correction. A two-tailed test was applied with voxel *p* < 0.001 and cluster *p* < 0.05. Subsequently, the differences in FC values identified in the PHN-anxiety group were transformed using Fisher's Z-transformation and then correlated with each patient's VAS and HAMA scores using Spearman correlation analysis.

Data from the mice were expressed as mean ± SEM. For normally distributed data, independent samples t-test or one-way ANOVA followed by LSD multiple comparison tests were used. If normality and homogeneity were not satisfied, the Mann-Whitney test, Kruskal-Wallis one-way ANOVA (followed by Dunn's test), or Wilcoxon pairwise test were performed. Two-way repeated-measures ANOVA was used to compare multiple groups under two testing conditions when appropriate (followed by Fisher's LSD multiple comparisons test). All analyses were performed using the two-tailed method with a significance threshold of *p* < 0.05.

## Results

### Part I Clinical fMRI research

#### Demographic and clinical characteristics

A total of 41 PHN-anxiety comorbidity patients and 40 age-, gender-, and education-matched HCs were included in this study. The HAMA scores of the patients with PHN-anxiety comorbidity patients were significantly higher than those of the HCs. The demographic and clinical information of all participants is detailed in Supplementary Table 1.

#### VBM analysis

**Figure 1:**
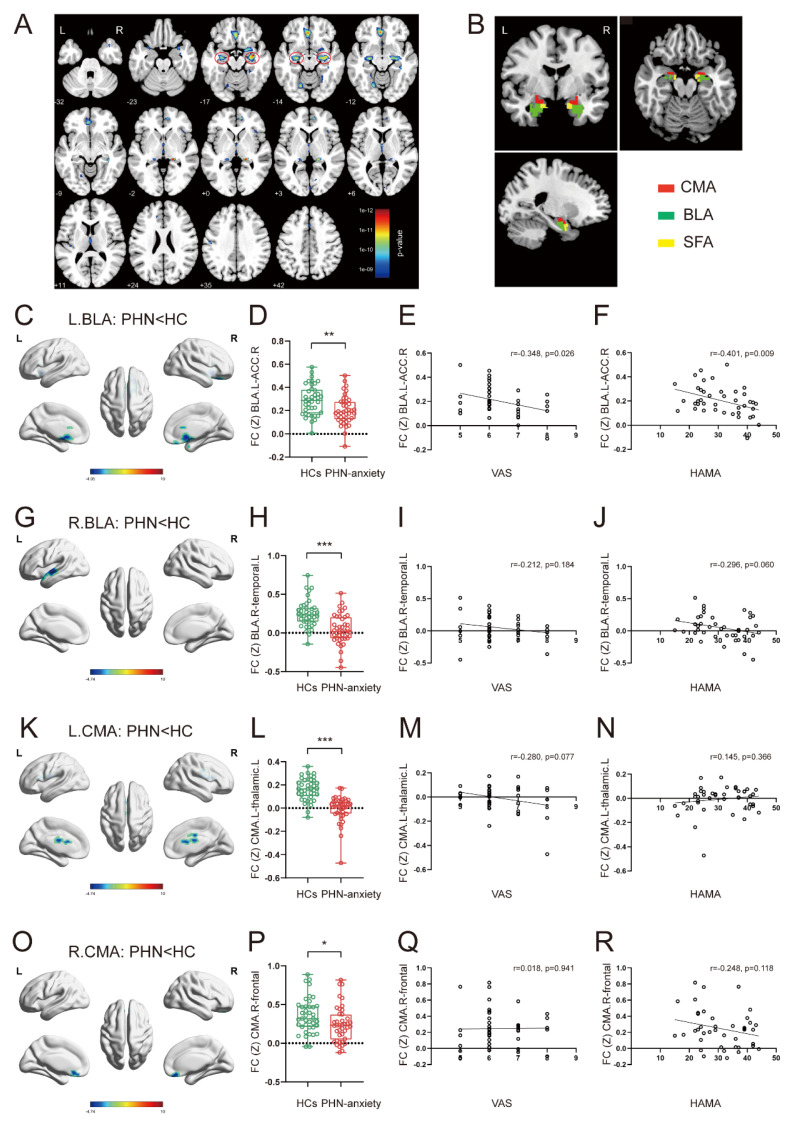
** Structural and functional changes in the amygdala in PHN-anxiety comorbidity patients compared to HCs.** (A) Compared to HCs, PHN-anxiety comorbidity patients had lower GM volumes in brain areas such as the bilateral amygdala/hippocampus, middle frontal gyrus, and fusiform gyrus (*p* < 0.05, FWE-corrected, k > 100 voxels). The most substantial reduction in GM volume was observed in the bilateral amygdala/hippocampus. (B) Seeds for the connectivity analyses correspond to the three subregions of the amygdala. (C) When the left BLA was used as a seed point, brain areas in PHN-anxiety had significantly reduced FC than in HC. (D) Comparison of FC values between the two groups of subjects (t = 3.137,* p* = 0.002). (E-F) Correlation of FC values with VAS and HAMA scores. (G) When the right BLA was used as a seed point, brain areas in PHN-anxiety comorbidity had significantly reduced FC than in HCs. (H) Comparison of FC values between the two groups of subjects (t = 4.799, *p* < 0.001). (I-J) Correlation of FC values with VAS and HAMA scores. (K-L) When the left CMA was used as a seed point, brain areas in PHN-anxiety had significantly reduced FC than in HC (t = 7.239, *p* < 0.001). (M-N) Correlation of FC values with VAS and HAMA scores. (O) When the right CMA was used as a seed point, brain areas in PHN-anxiety patients had significantly reduced FC than in HCs. (P) Comparison of FC values between the two groups of subjects (t = 2.215, *p* = 0.029). (Q-R) Correlation of FC values with VAS and HAMA scores. BLA, basolateral amygdala; CMA, central medial amygdala; SFA, superficial amygdala. (*p < 0.05, **p < 0.01, ***p < 0.001).

Compared with HCs, GM volumes were significantly reduced in the bilateral amygdala, hippocampus, fusiform gyrus, left middle frontal gyrus, lingual gyrus, thalamus, and middle cingulate gyrus in PHN-anxiety patients (Supplementary Table 2 and Fig. 1A). And the volumes of voxels in the amygdala and hippocampus was the highest compared to other regions.

#### The FC analysis based on seeds

The PHN-anxiety patients exhibited decreased FC between the left BLA and the right anterior cingulate cortex/prefrontal cortex compared with the HCs (Fig. 1C, D). Additionally, decreased FC between the right BLA and the left temporal lobe was observed in the PHN-anxiety group (Fig. 1G, H). Furthermore, the PHN-anxiety patients exhibited decreased FC between the left CMA and the left thalamus (Fig. 1K, L), as well as reduced FC between the right CMA and the bilateral frontal lobe compared with HCs (Fig. 1O, P) (Supplementary Table 3). No differences in the FC were found between the PHN-anxiety patients and HCs when SFA was used as seed.

#### Correlations between FC and clinical characteristics

We found that FC *Z* scores in the left BLA and the right ACC/prefrontal cortex of PHN-anxiety patients were negatively correlated with VAS (r = -0.348, *p* = 0.026) and HAMA scale (r = -0.401, *p* = 0.009) scores (Fig. 1E, F). There were no significant correlations between FC changes in other amygdala subregions and clinical features in PHN-anxiety patients (Fig. 1I-J, M-N, Q-R).

### Part II Animal research

#### RTX treatment led to PHN comorbid anxiety-like behaviors

**Figure 2:**
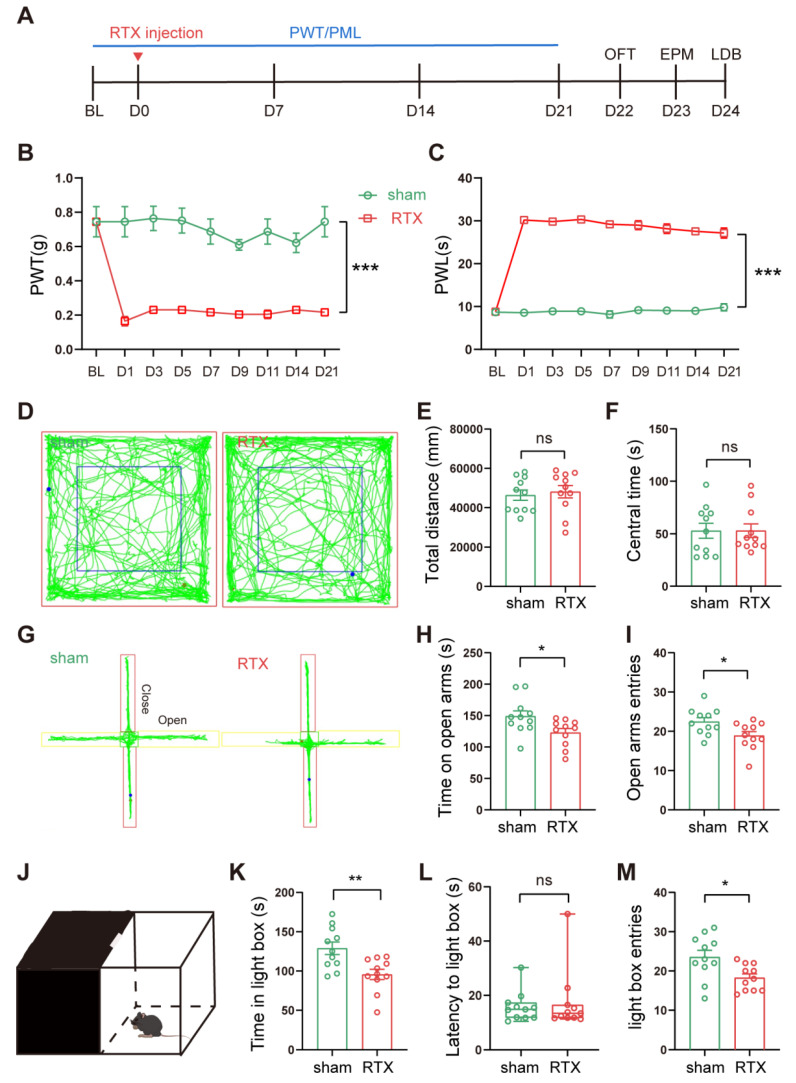
** Intraperitoneal injection of RTX leads to PHN comorbid anxiety-like behaviors.** (A) Intraperitoneal injection of RTX and experimental timeline. Mechanical threshold (B) and the thermal paw withdrawal latency (C) in sham and RTX mice (two-way ANOVA, F(8, 56) = 58.47, *p* < 0.0001; n = 11 mice/group). (D) Representative trace of OFT in sham mice and RTX mice. Statistical analysis of total distance traveled (E) and center time (F) in the OFT of mice 3 weeks after solvent or RTX injection (total distance traveled t = 0.439, *p* = 0.672; center time t = 0.011, *p* = 0.991). (G) Representative diagrams of the EPM in sham and RTX mice. (H) Time spent in the open arm (t = 2.423, *p* = 0.025) and (I) number of entries into the open arm (t = 2.433, *p* = 0.024) in the EPM test. (J) Schematic representation of the LDB experiment. (K) Time spent in the light box in sham and RTX mice (t = 3.232, *p* = 0.004), (L) latency to enter the light box (U = 53, *p* = 0.652), and (M) number of entries into the bright box (t = 2.689, *p* = 0.014) (n = 11 mice/group). (*p < 0.05, **p < 0.01, ***p < 0.001).

Von-Frey and Hargreaves's experiments were performed at different time points before and after a single intraperitoneal injection of RTX to assess the PWT and PWL of the mice, respectively. The anxiety behaviors of the mice were assessed by OFT, EPM, and LDB (Fig. 2A). After intraperitoneal injection of RTX, mice showed decreased PWT and increased PWL compared to mice injected with vehicle only (Fig. 2B, C). Three weeks after the RTX injection, the mice were tested for anxiety behaviors sequentially. In the EPM, compared to the sham group, the number of entries and the time stayed in the open arm of RTX mice were reduced (Fig. 2G-I). In the LDB, the time stayed in the lightbox and the number of entries to the lightbox was reduced in RTX mice (Fig. 2J-M). There was no significant difference in the total distance and the time in the center between the sham and the RTX mice in the OFT (Fig. 2D-F), indicating that the RTX model did not affect the motility of mice. In conclusion, these data suggest that RTX mice displayed PHN comorbid anxiety-like behaviors after 3 weeks after RTX exposure.

#### BLA^Glu^ neurons are activated in PHN comorbid anxiety-like mice

**Figure 3:**
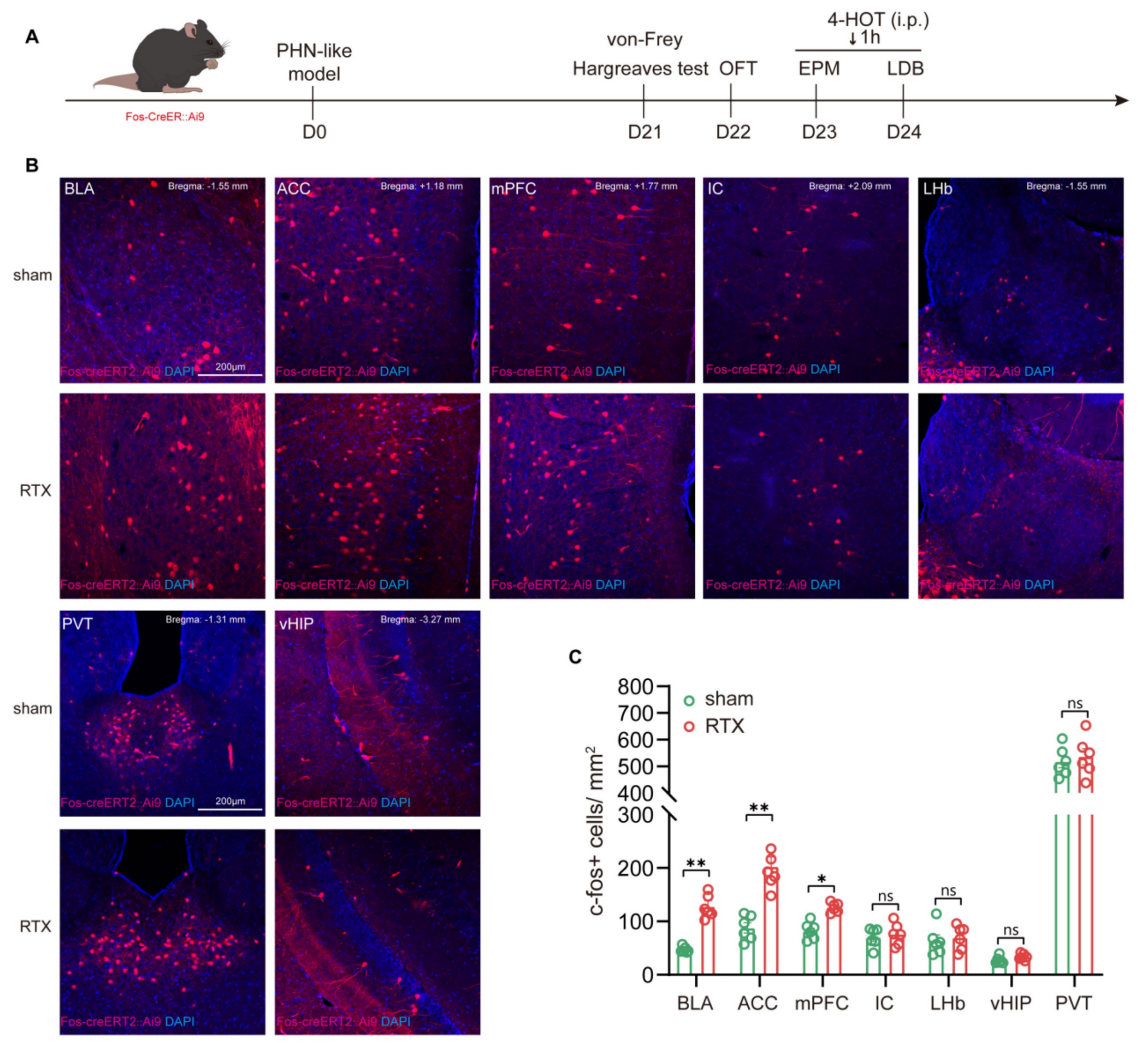
** PHN comorbid anxiety-like-dependent neuron labeling.** (A) Experimental flowchart. (B) Representative images showing Fos-CreER::Ai9 positive neurons in the BLA, ACC, mPFC, IC, LHb, PVT, and vHIP in Fos-CreER::Ai9 mice (scale bars, 200 μm). (C) Statistical plots of mean c-fos per square millimeter in different brain regions in the sham and PHN comorbid anxiety-like group (6 brain slices of 3 mice in each group, BLA: t = 4.707, *p* = 0.0012, ACC: t = 3.268, *p* = 0.0085, mPFC: t = 3.171, *p* = 0.010, IC: t = 0.547, *p* = 0.596, LHb: t = 0.416, *p* = 0.686, PVT: t = 0.494, *p* = 0.632, vHIP: t = 1.784, *p* = 0.105). (*p < 0.05, **p < 0.01).

To verify whether the brain regions showing volume differences in fMRI VBM analysis are activated in PHN comorbid anxiety-like mice, we used Fos-CreER::Ai9 mice to identify the activated neurons. 4-OHT was injected 1 hour before the behavioral tests on days 23 and 24 after modeling, respectively (Fig. 3A). Since 4-OHT remains effective for approximately 6 hours, the animals were placed in a quiet and familiar environment after the behavioral experiments to allow for drug metabolism. Based on the results of the fMRI and previous studies, we examined the Fos-CreER::Ai9 positive neurons in brain regions associated with pain and mood disorders, including the BLA, ACC, medial prefrontal cortex (mPFC), insular cortex (IC), lateral habenula (LHb), ventral hippocampus (vHIP), and paraventricular nucleus of the thalamus (PVT). We found that the number of Fos-CreER::Ai9 positive neurons was notably elevated only in the BLA, ACC, and mPFC of PHN comorbid anxiety-like mice as compared with the sham group, with the most prominent changes occurring in both the BLA and ACC brain areas (Fig. 3B, C).

**Figure 4:**
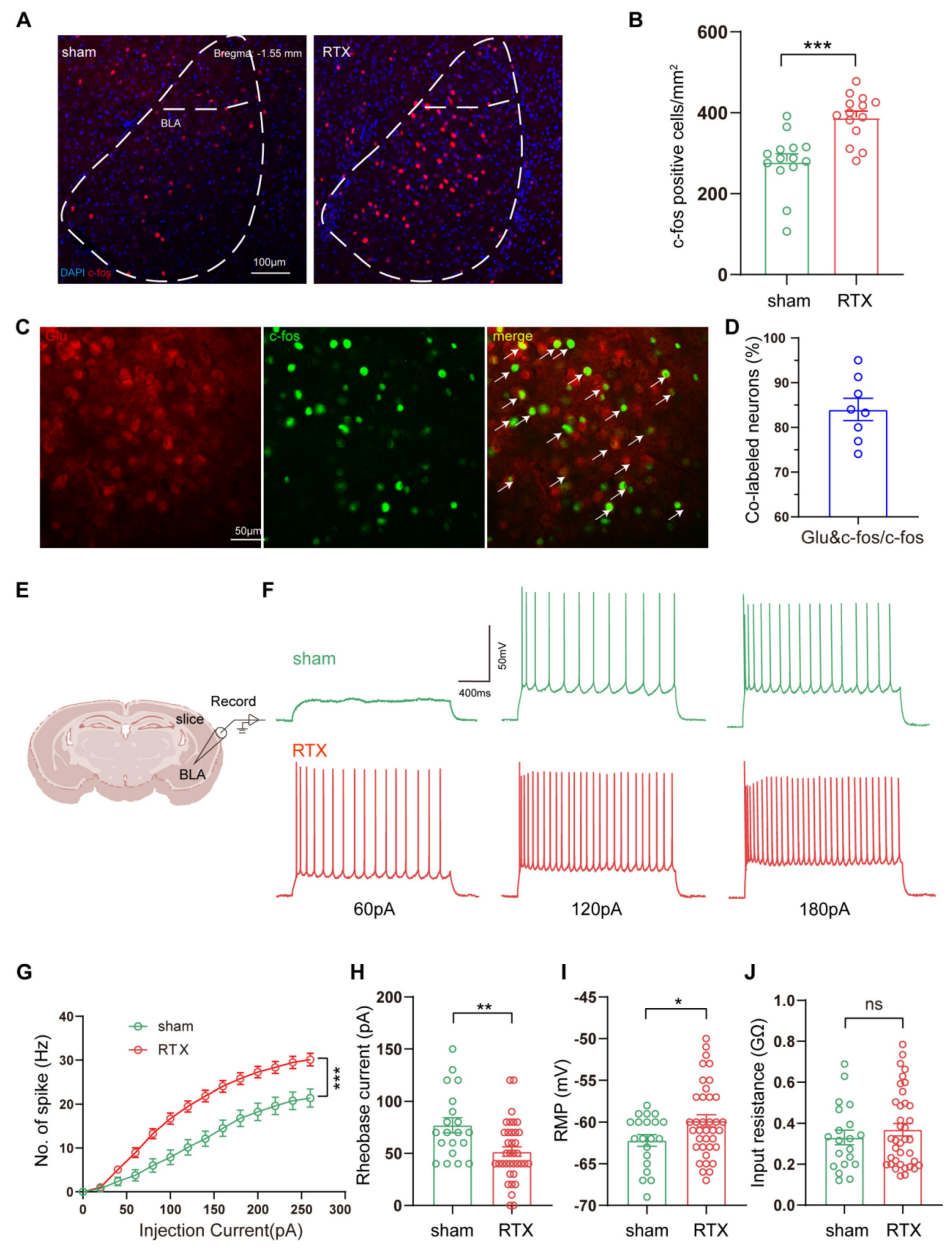
** Increased excitability of BLA^Glu^ neurons in PHN comorbid anxiety-like mice.** (A) Representative plots of immunohistochemical c-fos of BLA in sham vs PHN comorbid anxiety-like mice, scale bar 100 μm; (B) Statistical plots of mean c-fos per square millimeter of the BLA in both groups (14 brain slices of 4 mice in each group, t = 8.862, *p* < 0.001); (C) Representative plots of c-fos co-labeled with glutamate in the BLA brain region and (D) statistical graph (8 brain slices from 3 mice), scale bar 50 μm; (F) Representative graphs of action potential spikes recorded from BLA^Glu^ neurons in sham and PHN comorbid anxiety-like mice (E, F) and statistical graphs of the data (G) (6 mice in the sham group with a total of 20 neurons, and 7 mice in the PHN comorbid anxiety-like mice with a total of 37 neurons; F(13, 209) = 6.283, *p* < 0.0001). (H) Rheobase current (t = 3.091, *p* = 0.003), (I) RMP (t = 2.104, *p* = 0.040), and (J) Rin (t = 0.782, *p* = 0.437) in the sham and PHN comorbid anxiety-like group. (*p < 0.05, **p < 0.01, ***p < 0.001).

Combining the findings from fMRI and c-fos labeled positive neurons, we focused on the BLA to verify alteration in BLA neuronal activity under PHN comorbid anxiety-like condition by IHC. Compared with the sham group, a significant increase in c-fos-positive neurons was observed in the BLA (Fig. 4A, B). Given that previous studies have shown the BLA is dominated by glutamatergic neurons 35, we co-stained c-fos with glutamate and found that the rate of co-labeling was 84.01% (Fig. 4C, D). Based on the above data, we explored whether PHN-anxiety comorbidity alters the excitability of BLA^Glu^ neurons by recording the active and passive membrane properties of BLA^Glu^ neurons using whole-cell patch clamp recording *in vitro*. We discovered that when BLA neurons of PHN comorbid anxiety-like mice were subjected to a series of depolarizing stimuli (0 - 280 pA at 10 pA increments), their firing rates were significantly higher than those recorded in the sham group (Fig. 4E-G). In addition, the resting membrane potential (RMP) of the PHN comorbid anxiety-like mice was depolarized, the rheobase current was considerably lower than that of the controls, and there was no significant change in the Rin (Fig. 4H-J). The above studies demonstrated increased excitability of BLA^Glu^ neurons in PHN comorbid anxiety-like mice.

#### Manipulation of BLA^Glu^ neuronal activity regulated pain and anxiety behaviors in PHN comorbid anxiety-like mice

**Figure 5:**
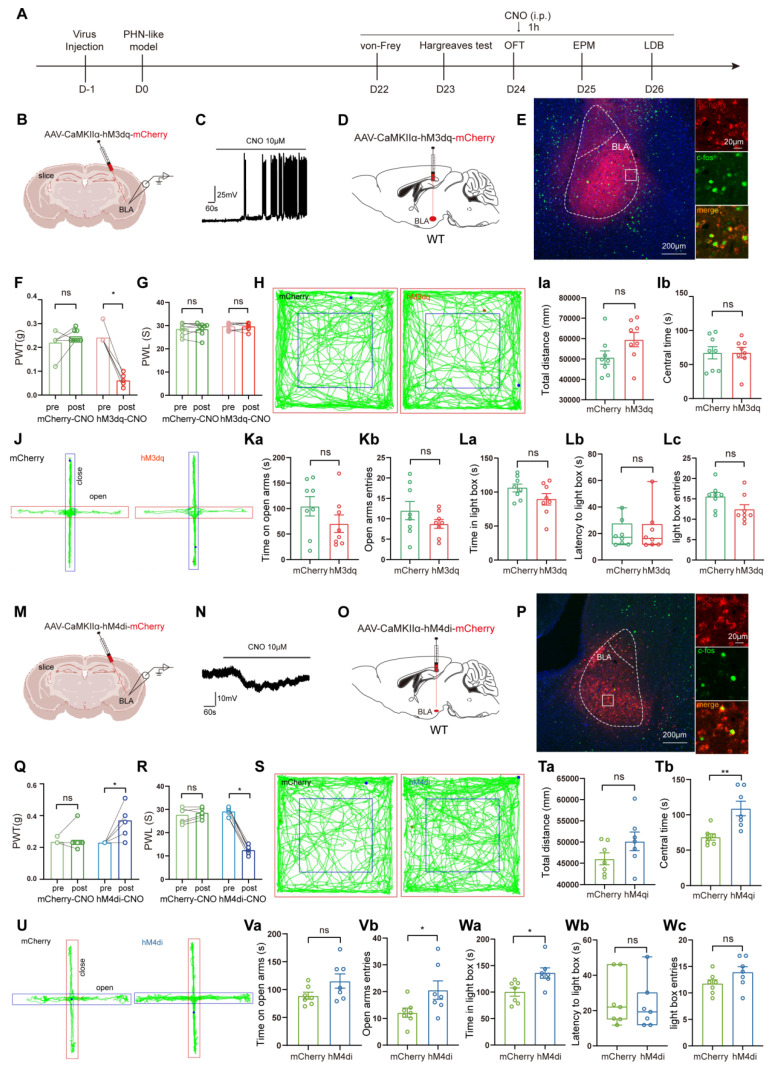
** Chemogenetic activation or inhibition of BLA^Glu^ neurons alters pain and anxiety-like behaviors in PHN comorbid anxiety-like mice.** (A) Experimental flowchart of chemogenetic activation. (B) Schematic diagram of AAV-CaMKIIα-hM3dq-mCherry virus injected in BLA and *in vitro* electrophysiology. (C) Whole-cell current clamp recordings of BLA show that CNO perfusion resulted in depolarization of BLA neurons expressing hM3dq. (D) Schematic of chemogenetic activation of BLA^Glu^ neurons in PHN comorbid anxiety-like mice and (E) representative chart of viral expression with a scale bar of 200 μm; white box magnified area with a scale bar of 20 μm. (F) Mechanical thresholds after stereotactic injection of control or hM3dq virus and intraperitoneal injection of CNO in PHN comorbid anxiety-like mice (Comparing before and after CNO injection, *p* was 0.250 and 0.008 in mCherry or hM3dq-mCherry group, respectively) and (G) latency of thermal paw withdrawal (Comparing before and after CNO injection, *p* was 0.609 and > 0.999 in mCherry or hM3dq-mCherry group, respectively) (n = 8 mice/group). (H) Representative plots of OFT trace in mCherry or hM3dq-mCherry group after CNO injection. (Ia) Total distance traveled (t = 1.834, *p* = 0.089) and (Ib) time in the central region (t = 0.012, *p* = 0.991) of the OFT in both groups of mice after CNO injection (n = 8 mice/group). (J) Representative plots of exploration trace in the EPM in mCherry or hM3dq-mCherry group after CNO injection. (Ka) Time spent in the open arm of the EPM (t = 1.333, *p* = 0.204) and (Kb) number of times entry the open arm (t = 1.301, *p* = 0.214) in both groups after injection of CNO. In the LDB, (La) both groups of mice spent time in the lightbox (t = 1.627, *p* = 0.126), (Lb) latency to enter the lightbox (U = 30, *p* = 0.878), and (Lc) number of times entering the lightbox (t = 1.936, *p* = 0.073) (n = 8 mice/group). (M) Schematic diagram of electrophysiology after injection of AAV-CaMKIIα-hM4di-mCherry virus in the BLA. (N) BLA whole-cell membrane clamp recordings show that CNO perfusion resulted in hyperpolarization of BLA neurons expressing hM4di. (O) Schematic of chemical genetic inhibition of BLA^Glu^ neurons in PHN comorbid anxiety-like mice and (P) Representative chart of viral expression, scale bar 200 μm, white box magnified area scale bar 20 μm. (Q) Mechanical thresholds of PHN comorbid anxiety-like mice after injection of control or hM4di viruses and intraperitoneal injection of CNO (Comparing before and after CNO injection, *p* > 0.999 and *p* = 0.031 in mCherry or hM4di-mCherry group, respectively) and (R) latency of thermal paw withdrawal (Comparing before and after CNO injection, *p* = 0.187 and *p* = 0.016 in mCherry or hM4di-mCherry group, respectively) (n = 7 mice/group). (S) Representative plots of OFT trace in mCherry or hM4di-mCherry after CNO injection. (Ta) Total distance (t = 1.569, *p* = 0.143) and (Tb) time in the central region (t = 3.572, *p* = 0.004) of the OFT in both groups of mice after CNO injection (n = 7 mice/group). (U) Representative plots of exploration trace in the EPM of PHN comorbid anxiety-like mice with mCherry or hM4di-mCherry and injection of CNO. (Va) The time spent in the open arm of the EPM (t = 1.837, *p* = 0.091) and (Vb) the number of times entered the open arm (t = 2.189, *p* = 0.049) in both groups after CNO injection. In the LDB experiment, (Wa) the duration of stay in the lightbox (t = 2.844, *p* = 0.015), (Wb) latency to enter the lightbox (U = 23, *p* = 0.877), and (Wc) number of entries into the lightbox (t = 1.633, *p* = 0.128) in both groups of mice (n = 7 mice/group). (*p < 0.05, **p < 0.01, ***p < 0.001).

Since BLA^Glu^ neurons were significantly activated in PHN comorbid anxiety-like mice, we sought to determine whether the activation of these neurons could aggravate pain and anxiety-like behaviors in PHN comorbid anxiety-like mice. To accomplish this, we injected AAV-CaMKIIα-hM3dq-mCherry into the BLA of PHN comorbid anxiety-like mice with AAV-CaMKIIα-mCherry viruses a control (Fig. 5A, B). To verify the virus's effectiveness, we performed *in vitro* electrophysiology recordings on virus-transfected BLA neurons, and we observed a depolarization accompanied by a significant increase in spontaneous firing upon perfusion with CNO (10 μM) (Fig. 5B, C). IHC demonstrated that the majority of the BLA mCherry-positive neurons were co-labeled with c-fos (Fig. 5D, E), further confirming successful transfection and manipulation. Activation of BLA^Glu^ neurons *in vivo* by intraperitoneal injection of CNO three weeks after the viral transfection resulted in a marked reduction in PWT (Fig. 5F) in hM3dq-treated mice. However, PWL (Fig. 5G), as well as anxiety behaviors (Fig. 5H-L), did not significantly change. Next, we asked whether inhibition of BLA^Glu^ neurons could alleviate pain and anxiety-like behavior in PHN comorbid anxiety-like mice. Using the same strategy, we injected AAV-CaMKIIα-hM4di-mCherry into the BLA (Fig. 5M). *In vitro* electrophysiological recording demonstrated that CNO perfusion resulted in hyperpolarization of membrane potentials in BLA neurons (Fig. 5N). IHC revealed that only a tiny proportion of the BLA mCherry-positive neurons were co-labeled with c-fos (Fig. 5O, P), confirming the effectiveness of the virus. The PWT and PWL were relieved in the hM4di-treated mice after the injection of CNO (Fig. 5Q, R). Furthermore, compared with the mCherry group, hM4di-treated mice spent more time in the center of the OFT without any change in total distance traveled, suggesting that inhibition of BLA^Glu^ did not affect the motion function (Fig. 5S, T). The hM4di-treated mice also showed increased entries and longer durations in the open arms in EPM (Fig. 5U, V), along with a greater willingness to stay in the lightbox in LDB (Fig. 5W).

We next asked whether optogenetic stimulation of BLA^Glu^ neurons exacerbates pain and anxiety-like behaviors in PHN comorbid anxiety-like mice. To answer this question, we injected AAV-CaMKIIα-ChR2-mCherry or AAV-CaMKIIα-mCherry viruses into the BLA, followed by bilateral implantation of optical fibers. RTX model was established the day after virus injection (Fig. S1A). We first verified the efficiency of AAV-CaMKIIα-ChR2-mCherry by recording AP induced by 470 nm blue light at 5 Hz using *in vitro* electrophysiology (Fig. S1B, C). Then, we performed *in vivo* optogenetic experiments. Optical activation of BLA^Glu^ neurons in RTX mice led to further exacerbation of mechanical hypersensitivity, as indicated by a reduced PWT. In terms of anxiety-like behavior, the results of the OFT and EPM showed no statistical difference between the two groups (Fig. S1H-M). Next, we injected either AAV-CaMKIIα-eNPHR-mCherry virus or control virus into the BLA and conducted *in vitro* electrophysiological recording to confirm that the eNPHR-infected BLA neurons could be suppressed by 590 nm yellow laser (Fig. S1D). After three weeks of viral transfection, the pain and anxiety-like behaviors of the RTX mice were evaluated upon yellow light stimulation. The results showed that the PWT of the eNPHR-treated mice was notably elevated, indicating significant pain relief (Fig. S1G). In the OFT, the eNPHR-treated mice spent more time in the center, although the total distance traveled was not significantly different (Fig. S1N-P). Similarly, no significant difference was observed between the two groups in the EPM (Fig. S1Q-S). The above results suggest that specific activation of BLA^Glu^ neurons worsened mechanical pain in PHN comorbid anxiety-like mice but did not exacerbate anxiety-like behaviors while inhibiting the excitability of BLA^Glu^ neurons could effectively alleviate both pain and anxiety in PHN comorbid anxiety-like mice.

#### Elucidation of an excitatory BLA^Glu^-ACC circuit

**Figure 6:**
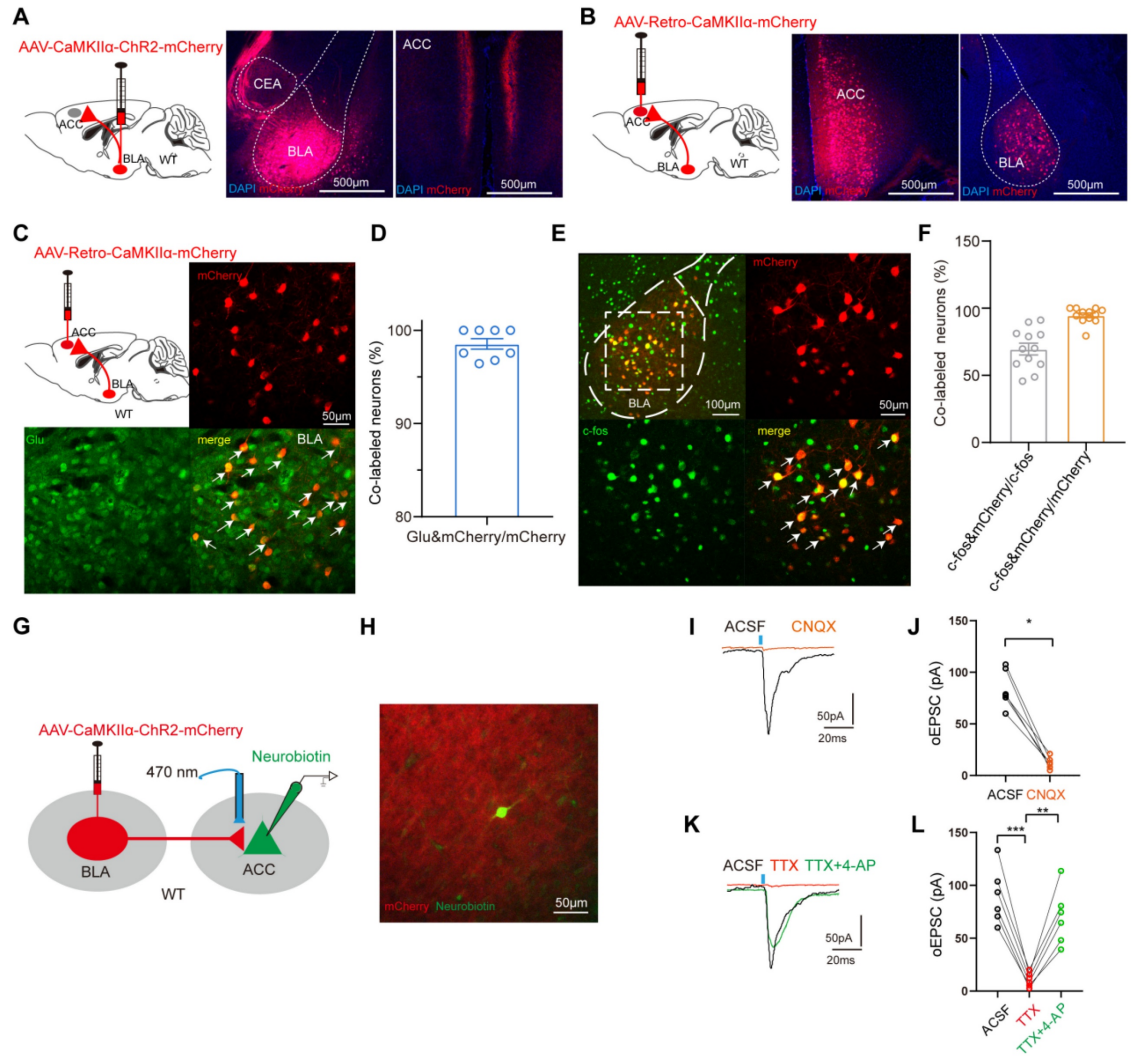
** Dissection of the BLA^Glu^-ACC circuit.** (A) Schematic of BLA virus tracing in WT mice (left), representative chart of ChR2 virus expressing in BLA (center), and example chart of BLA projection fibers in ACC (right), scale bar 500 μm. (B) Schematic of ACC injected retrograde virus tracing in WT mice (left), representative chart of viral injection sites (center), and virus expression in BLA (right), scale bar 500 μm. (C) Representative chart of BLA neurons co-labeled with glutamate from ACC^Glu^ retrograde tracing and (D) quantitative analysis (n = 3 mice, total of 8 brain slices), scale bar 50 μm. (E) Representative map of BLA neurons co-labeled with c-fos in retrograde labeling of BLA neurons from ACC pyramidal neurons in PHN comorbid anxiety-like mice, scale bar 100 μm; the white box denotes the zoomed-in BLA area shown, scale bar 20 μm and (F) graph of the analysis of the co-labeled rate of the two (c-fos co-stained with mCherry in about 69.59% of all c-fos+ neurons; c-fos co-stained with mCherry in about 94.69% of all mcherry+ neurons) (n = 4 mice, total of 12 brain slices). (G) Optogenetically activated virus was injected in the BLA and electrophysiologic recordings were performed on ACC pyramidal neurons. (H) Schematic of BLA projecting terminal and Neurobiotin-labeled neurons in ACC. (I) The oEPSC recorded in ACC was blocked by CNQX and (J) statistical graph (*p* = 0.0003) (n = 3 mice, 6 neurons in total). (K) oEPSC was completely blocked by TTX and rescued with 4-AP, and (L) corresponding statistical graphs (ACSF vs TTX *p* = 0.031, TTX vs TTX+4-AP *p* = 0.001) (n = 3 mice, 6 neurons in total). (*p < 0.05, **p < 0.01, ***p < 0.001).

As mentioned above, fMRI analysis revealed a reduction FC between BLA and ACC, and modulation of the excitability of BLA^Glu^ neurons altered pain and anxiety-like behaviors in PHN comorbid anxiety-like mice. These data raised another question: do BLA^Glu^ neurons function in concert with the ACC region? To examine the anatomical arrangement of BLA^Glu^ neuron projections, we first injected the AAV-CaMKIIα-ChR2-mCherry into the BLA and discovered abundant projections in layers II/III of the ACC (Fig. 6A). Subsequently, retrograde labeling was performed by injecting AAV-Retro-CaMKIIα-mCherry virus into the ACC, which labeled BLA^Glu^ neurons projecting to ACC (Fig. 6B). A majority of these retrogradely labeled neurons were co-labeled with glutamate (Fig. 6C, D), and accounting for about 69.59% of the c-fos positive neurons in PHN comorbid anxiety-like mice (Fig. 6E, F). These results imply the BLA^Glu^-ACC^Glu^ circuit may play a significant role in PHN comorbid anxiety-like behaviors. Considering that the BLA projects densely to ACC II/III layers and the fact that layers II/III of the ACC serve as primarily receiving layers reported by previous studies 25, 36, we performed *in vitro* optogenetic manipulation and electrophysiological recordings from neurons located in II/III layers in ACC to verify further the functional connectivity of this circuit (Fig. 6G). K^+^ electrode internal fluid with Neurobiotin was applied for the recordings, and only neurons surrounded by mCherry-labeled projecting terminals confirmed by post-hoc immunofluorescent imaging were included for further analysis (Fig. 6H). The oEPSC was blocked by the AMPA receptor antagonist CNQX (100 μM) (Fig. 6I, J), confirming the presence of excitatory synaptic transmission within the BLA^Glu^-ACC circuit. In addition, oEPSC could be inhibited by TTX (1 μM) and later recovered with TTX and the 4-AP (1 mM), suggesting a monosynaptic connection between BLA^Glu^ and ACC (Fig. 6K, L). Taken together, these results provide solid evidence for the existence of excitatory monosynaptic transmission in the BLA^Glu^-ACC circuit.

#### The excitability of ACC glutamatergic neurons was enhanced in PHN comorbid anxiety-like mice

**Figure 7:**
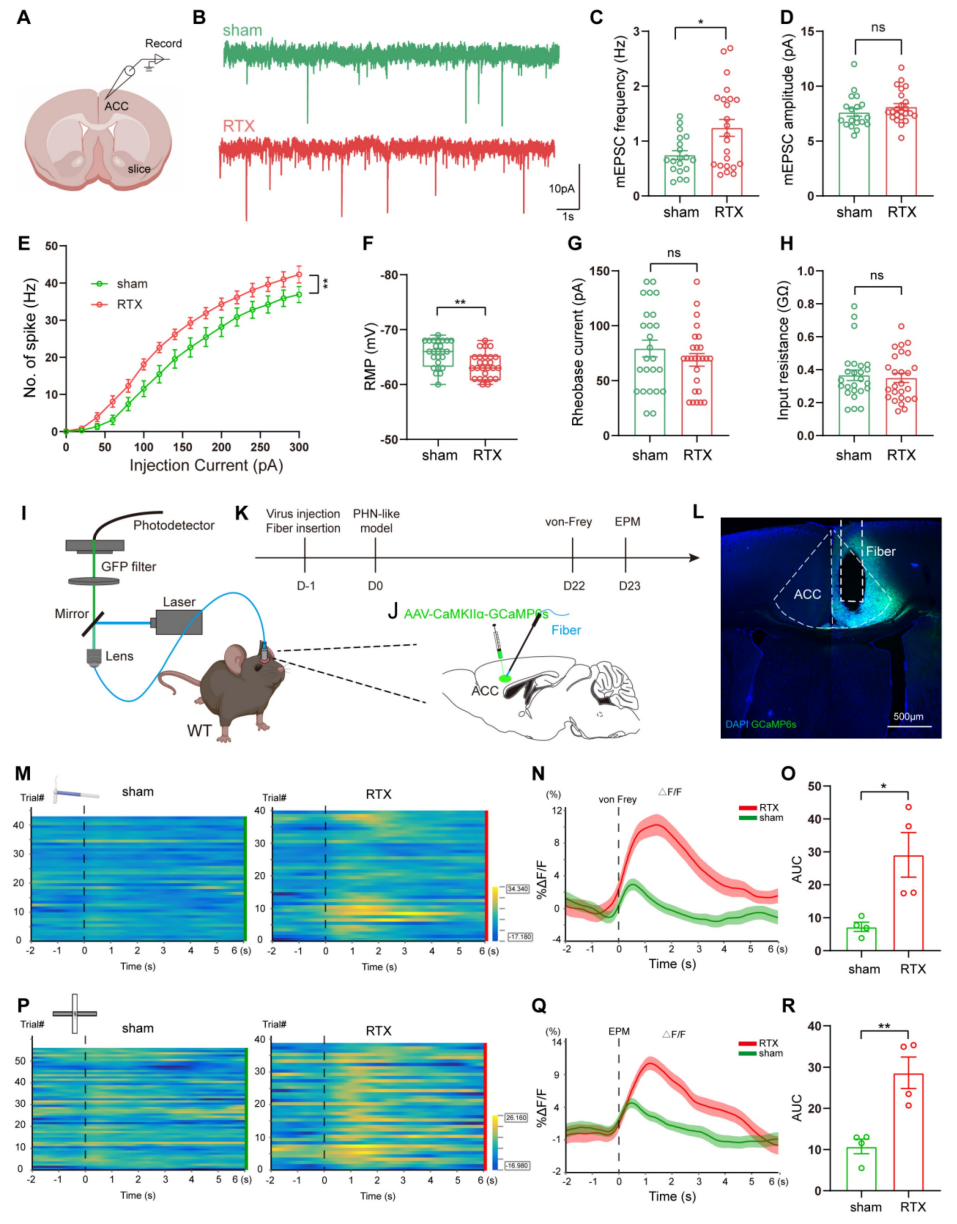
** Increased excitability of ACC glutamatergic neurons in PHN comorbid anxiety-like mice.** (A) Schematic diagram of *in vitro* electrophysiology of ACC slices. (B) Representative charts of mEPSC in sham and PHN comorbid anxiety-like group. (C) Comparison of mEPSC frequency (4 mice in the sham group, 19 neurons in total; 4 mice in the PHN comorbid anxiety-like group, 23 neurons in total; t = 2.697, *p* = 0.010) and (D) amplitudes in ACC about sham and PHN comorbid anxiety-like mice (4 mice in the sham group, 19 neurons in total; 4 mice in the PHN comorbid anxiety-like group, 23 neurons in total; t = 1.075,* p* = 0.289). (E) The statistical plot of action potential firing after electrical evocation (F (15, 248) = 2.609, *p* = 0.001; 6 mice in the sham group with a total of 25 neurons; 7 mice in the PHN comorbid anxiety-like group with a total of 26 neurons). (F) RMP of mice in the sham and PHN comorbid anxiety-like group (t = 3.277, *p* = 0.002), (G) Rheobase current (t = 1.093, *p* = 0.280), and (H) Rin (t = 0.382, *p* = 0.704). (I) Schematic of fiber optic recording of calcium signals, box enlargement for (J) virus injection, and fiber optic position. (K) Flowchart of calcium signal recording in sham and PHN comorbid anxiety-like mice. (L) Representative diagram of GCaMP6s virus expression and fiber-optic cannulation, scale bar 500 μm. (M) Heatmap of calcium during mechanical pain stimulation in sham and PHN comorbid anxiety-like mice. (N) Quantitative statistical plots of mean calcium signal and (O) mean fluorescent calcium response (t = 3.150, *p* = 0.020, n = 4 mice/group). (P) Heatmap of calcium signal changes in sham and PHN comorbid anxiety-like mice when moving from the closed arm into the open arm in EPM. (Q) Mean fluorescent calcium signal and (R) Mean fluorescent calcium response statistic plots (t = 4.252, *p* = 0.005, n = 4 mice/group). (*p < 0.05, **p < 0.01, ***p < 0.001).

To assess changes in the excitability of ACC pyramidal neurons of PHN comorbid anxiety-like mice, we performed *in vitro* electrophysiological recordings to evaluate the spike characteristics of II/III layer neurons in ACC (Fig. 7A). Excitatory pyramidal neurons were identified based on their regular spiking pattern 34. Miniature excitatory postsynaptic currents (mEPSC) were obtained after perfusing TTX (10 μM). We found that the frequency of mEPSC in PHN comorbid anxiety-like mice was significantly increased, with no significant change in amplitude (Fig. 7B-D), suggesting an enhanced presynaptic excitatory transmitter release in ACC. Compared to the sham group, PHN comorbid anxiety-like mice showed an increased spike frequency and a depolarized RMP, while there was no significant difference in rheobase current and Rin (Fig. 7E-H). Next, we injected AAV-CaMKII-GCaMP6s unilaterally into ACC and recorded the GCaMP6s signals of ACC glutamatergic neurons via fiber-photometry (Fig. 7I-L). Compared to the sham group, PHN comorbid anxiety-like mice showed a marked increase in Ca^2+^ fluorescence signal in response to painful stimulation and EPM test (Fig. 7M-R). In summary, these results indicated that neuronal excitability and presynaptic transmission were augmented in ACC glutamatergic neurons of PHN comorbid anxiety-like mice.

#### BLA^Glu^-ACC projections modulated pain and anxiety behavior in PHN comorbid anxiety-like mice

**Figure 8:**
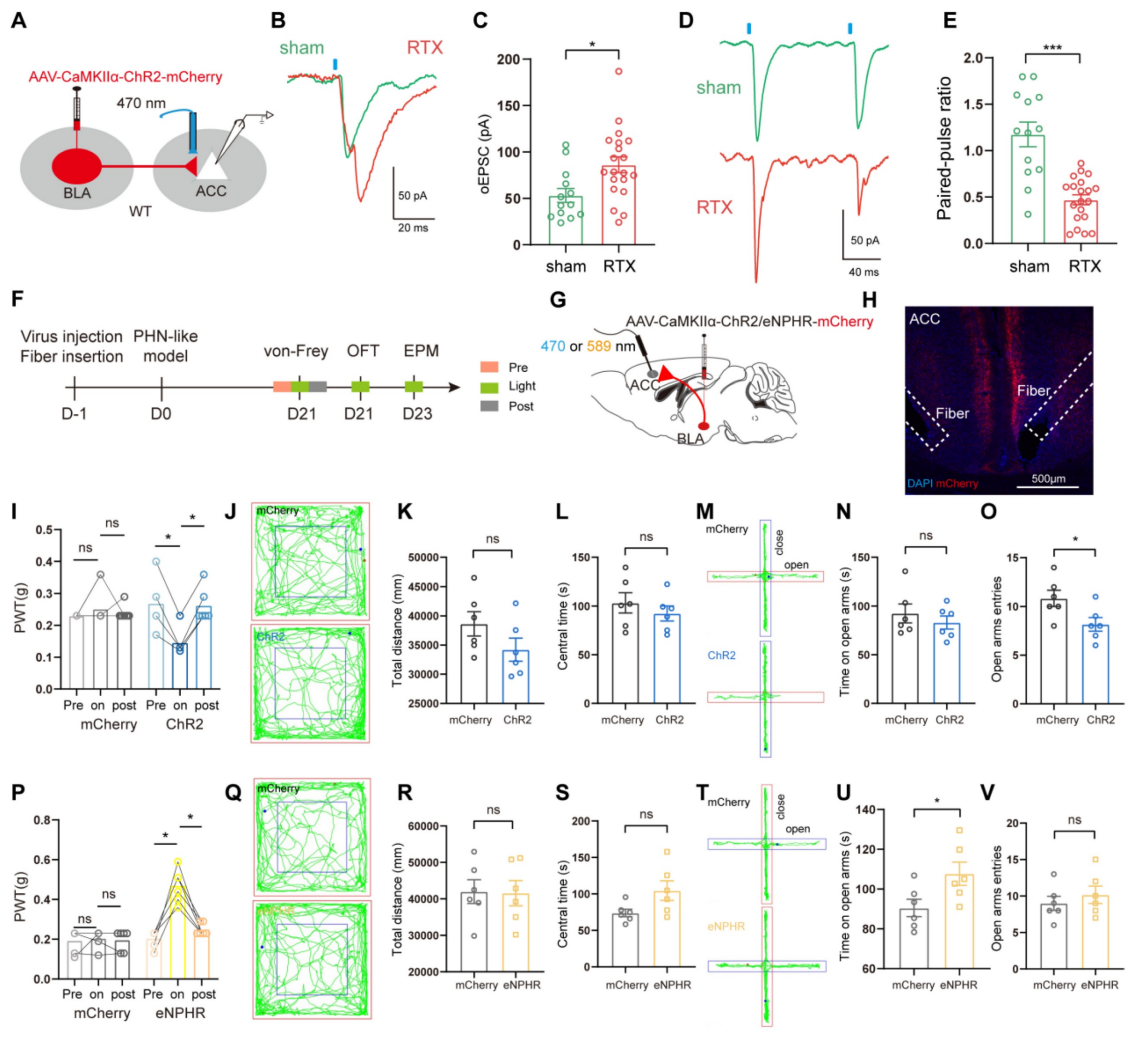
** Behavioral effects of optical regulation of BLA^Glu^-ACC projections in PHN comorbid anxiety-like mice.** (A) Schematic of virus injection and optical fiber implantation. (B) Representative plots of oEPSC in sham and PHN comorbid anxiety-like groups and (C) Statistical plots (5 mice in the sham group with 14 neurons; 5 mice in PHN comorbid anxiety-like group with 21 neurons; t = 3.034, *p* = 0.005). (D) Representative plots of PPR in the sham and the PHN comorbid anxiety-like group and (E) statistical plots (5 mice in the sham group, 14 neurons in total; 5 mice in the PHN comorbid anxiety-like group, 21 neurons in total; t = 5.622, *p* < 0.001). (F) Optogenetic flow chart. (G) Schematic diagram of BLA virus injection and ACC fiber implantation. (H) Representative diagram of virus expression and fiber optic implantation in ACC, scale bar: 500 μm. (I) The effects of optical activation of BLA^Glu^-ACC projections on pain threshold in the mCherry and the ChR2 group (blue-light induced in the mCherry group: pre vs on *p* > 0.999, on vs post* p* > 0.999; ChR2 group: pre vs on *p* = 0.031, on vs post *p* = 0.031) (n = 6 mice/group). (J) Representative plots of the action trace of the two groups in OFT, (K) statistical plots of total distance (t = 1.541, *p* = 0.154), and (L) time spent in the central region (t = 0.838, *p* = 0.421). (M) Representative plots of the action trace of the two groups in the EPM, with statistical plots of (N) time spent in the open arm (t = 0.791, *p* = 0.447) and (O) number of entries in the open arm (t = 2.446, *p* = 0.034). (P) The effects of optical inhibition of BLA^Glu^-ACC projections on pain threshold in the mCherry and the eHPHR group (yellow light-induced mCherry group: pre vs on *p* > 0.999, on vs post *p* > 0.999; eNPHR group: pre vs on *p* = 0.031, on vs post *p* = 0.031) (n = 6 mice/group). (Q) Representative plots of the trace of the two groups in OFT, (R) statistical plots of the total distance (t = 0.089, *p* = 0.931), and (S) time in the central region (t = 2.119, *p* = 0.060). (T) Representative plots of the trace of both groups in the EPM, (U) statistical plots of the time spent in the open arm (t = 2.361, *p* = 0.039), and (V) number of entries to the open arm (t = 0.759, *p* = 0.465). (*p < 0.05, **p < 0.01, ***p < 0.001).

To further investigate whether the enhanced presynaptic transmission of ACC pyramidal neurons in PHN comorbid anxiety-like mice was related to BLA^Glu^-ACC projections, AAV2/9-CaMKIIα-ChR2-mCherry was injected in BLA (Fig. 8A). *In vitro* electrophysiological results from ACC pyramidal neurons demonstrated that the amplitude of oEPSC in PHN comorbid anxiety-like mice was significantly larger than that of the sham group (Fig. 8B, C). Additionally, a reduced PPR was identified in these ACC neurons of PHN comorbid anxiety-like mice (Fig. 8D, E), suggesting an increased presynaptic release from the BLA^Glu^ neurons to ACC pyramidal neurons. We then examined the effects of optogenetic modulation of the BLA^Glu^-ACC projections in PHN comorbid anxiety-like mice. After injecting AAV2/9-CaMKIIα-ChR2/eNPHR-mCherry viruses or control viruses in bilateral BLA and implanting optical fibers in bilateral ACC (Fig. 8F-H), we found that activation of BLA^Glu^-ACC projections by blue light not only further exacerbated mechanical pain (Fig. 8I), but also decreased entries to the open arm in EPM in ChR2-treated mice (Fig. 8M-O). There was no difference in OFT between the two groups (Fig. 8J-L). In contrast, inhibition of the BLA^Glu^-ACC projections by yellow light effectively alleviated mechanical pain (Fig. 8P) and raised time in the open arms in EPM experiments in eNPHR-treated mice (Fig. 8T-V). In OFT experiments, no statistical differences were observed in either the total distance traveled or the time spent in the central in both groups (Fig. 8Q-S).

Furthermore, we reconfirmed the functional significance of BLA^Glu^-ACC projections in modulating pain and anxiety-like behaviors in RTX mice through chemogenetic manipulation. AAV2/9-CaMKIIα-hM3dq-mCherry or AAV2/9-CaMKIIα-mCherry was injected into BLA and microinjection tubes were implanted over ACC bilaterally (Fig. S2A-D). A substantial number of mCherry-labeled axon terminals were observed in the ACC (Fig. S2E). Pain and anxiety-like behaviors were assessed 30 minutes following the microinjection of CNO into ACC. The results showed that activation of the BLA^Glu^-ACC projections exacerbated the pain and anxiety-like behaviors in PHN mice (Fig. S2F-L). Next, we utilized AAV2/9-CaMKIIα-hM4di-mCherry to inhibit these projections in PHN mice and found that PWT and PWL were significantly rescued in hM4di-treated mice (Fig. S2M, N). In the OFT, the hM4di-treated mice spent more time in the center compared to the controls (Fig. S2O, P), and the entries in the open arm along with the time spent in the open arm increased in EPM (Fig. S2Q, R). However, there was no difference in the LDB test in the two groups (Fig. S2S). Overall, the above results suggest that excitatory synaptic transmissions of the BLA^Glu^- ACC circuit were enhanced, and projections from BLA^Glu^ neurons to ACC were required for regulating PHN-related pain and anxiety-like behaviors.

#### BLA^ACC^ neurons were involved in pain and anxiety-like behaviors in PHN comorbid anxiety-like mice

**Figure 9:**
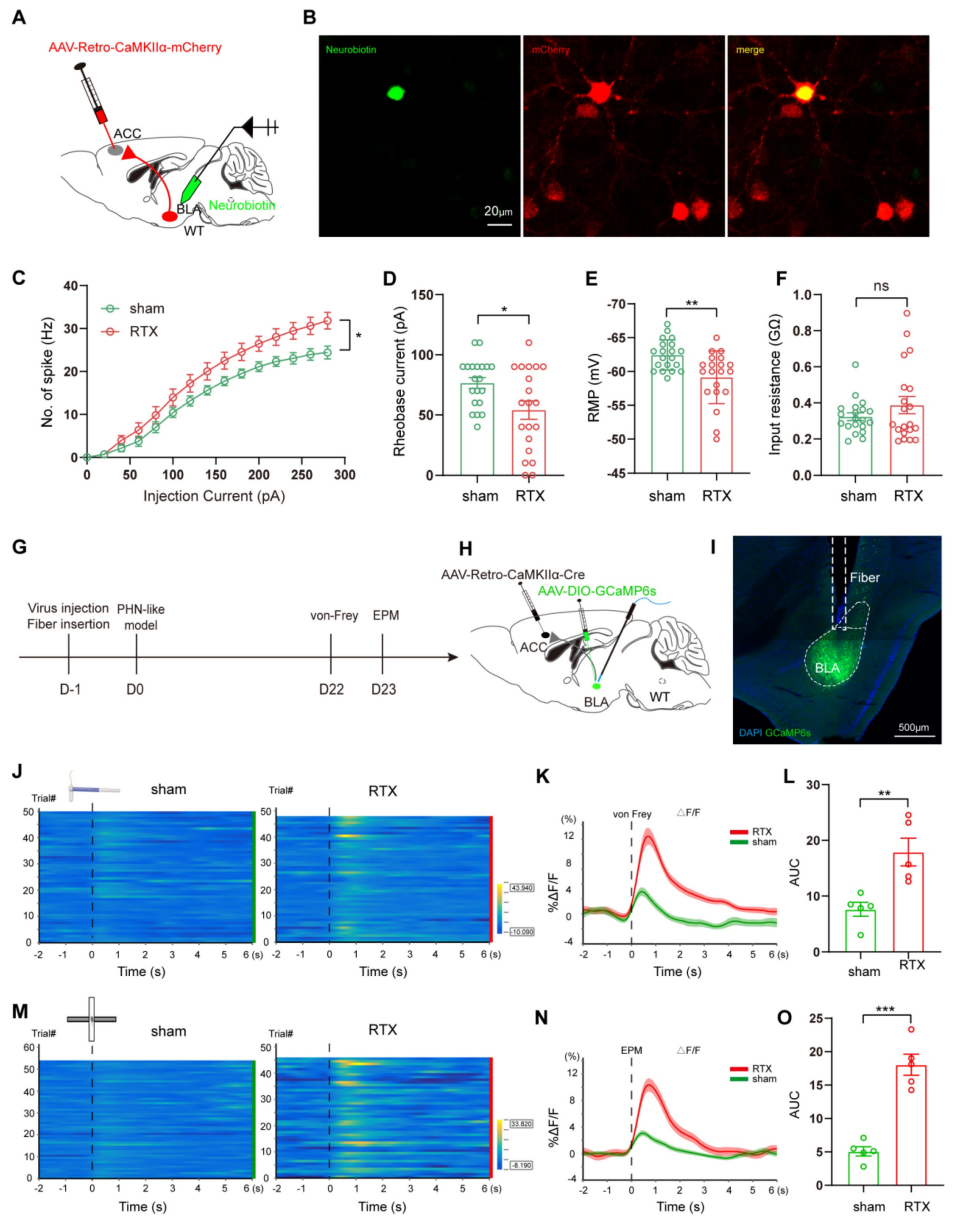
** Increased excitability of BLA^ACC^ neurons in PHN comorbid anxiety-like mice.** (A) Schematic of ACC injected with AAV/Retro-CaMKIIα-mCherry virus and whole-cell membrane clamped on BLA mCherry+ neurons. (B) Schematic of Neurobiotin-labeled neurons (left), mCherry+ neurons (center), and co-labeled of both (right), scale bar 20 μm. (C) Comparison of spikes in BLA^ACC^ neurons in mice of the sham and PHN comorbid anxiety-like group (5 mice in the sham group with a total of 20 neurons; 6 mice in the PHN comorbid anxiety-like group with a total of 20 neurons; F(14, 266) = 4.997, *p* < 0.0001), (D) comparison of Rheobase (t = 2.518, *p* = 0.016), (E) RMP (t = 3.29, *p* = 0.002) and (F) Rin (t = 1.230, *p* = 0.226). (G) Flowchart of calcium signal recording in sham and PHN comorbid anxiety-like mice. (H) Schematic of virus injection and fiber optic position. (I) Representative diagram of GCaMP6s virus expression and fiber-optic cannulation, scale bar 500 μm. (J) Heatmap of calcium during mechanical pain stimulation in sham and PHN comorbid anxiety-like mice. (K) Quantitative statistical plots of mean calcium signal and (L) mean fluorescent calcium response (t = 3.689, *p* = 0.006, n = 5 mice/group). (M) Heatmap of calcium signal changes in sham and PHN comorbid anxiety-like mice when moving from the closed arm into the open arm in EPM. (N) Mean fluorescent calcium signal and (O) Mean fluorescent calcium response statistic plots (t = 7.548, *p* < 0.001, n = 5 mice/group). (*p < 0.05, **p < 0.01, ***p < 0.001).

Since BLA^Glu^ neurons play a crucial role in modulating PHN-related pain and anxiety behaviors via the BLA^Glu^-ACC projections, we further investigated the contribution of BLA^ACC^ neurons in PHN comorbid anxiety-like mice. To selectively label the BLA^ACC^ neurons, we injected AAV/Retro-CaMKIIα-mCherry into the ACC. After 3 weeks of viral transfection, *in vitro* electrophysiological recordings were performed to assess the properties of the mCherry+ neurons in BLA (Fig. 9A, B). Compared to those of the sham group, BLA^ACC^ neurons from PHN comorbid anxiety-like mice exhibited increased spikes (Fig. 9C), along with depolarized RMP and more significant rheobase current (Fig. 9D, E), while there was no difference in Rin (Fig. 9F). We also assessed calcium signals of BLA^ACC^ neurons during nociceptive stimuli and anxiety-like behaviors. To do this, we injected AAV/Retro-CaMKIIα-Cre into the ACC, AAV-DIO-GCaMP6s into the BLA, and fiber optics were implanted in BLA (Fig. 9G-I). We noticed that the Ca^2+^ activity of BLA^ACC^ neurons from PHN comorbid anxiety-like mice was significantly higher than the sham group in pain stimulation and the EPM test (Fig. 9J-O). Collectively, the above results demonstrated increased neural activity of BLA^ACC^ neurons in PHN comorbid anxiety-like mice.

**Figure 10:**
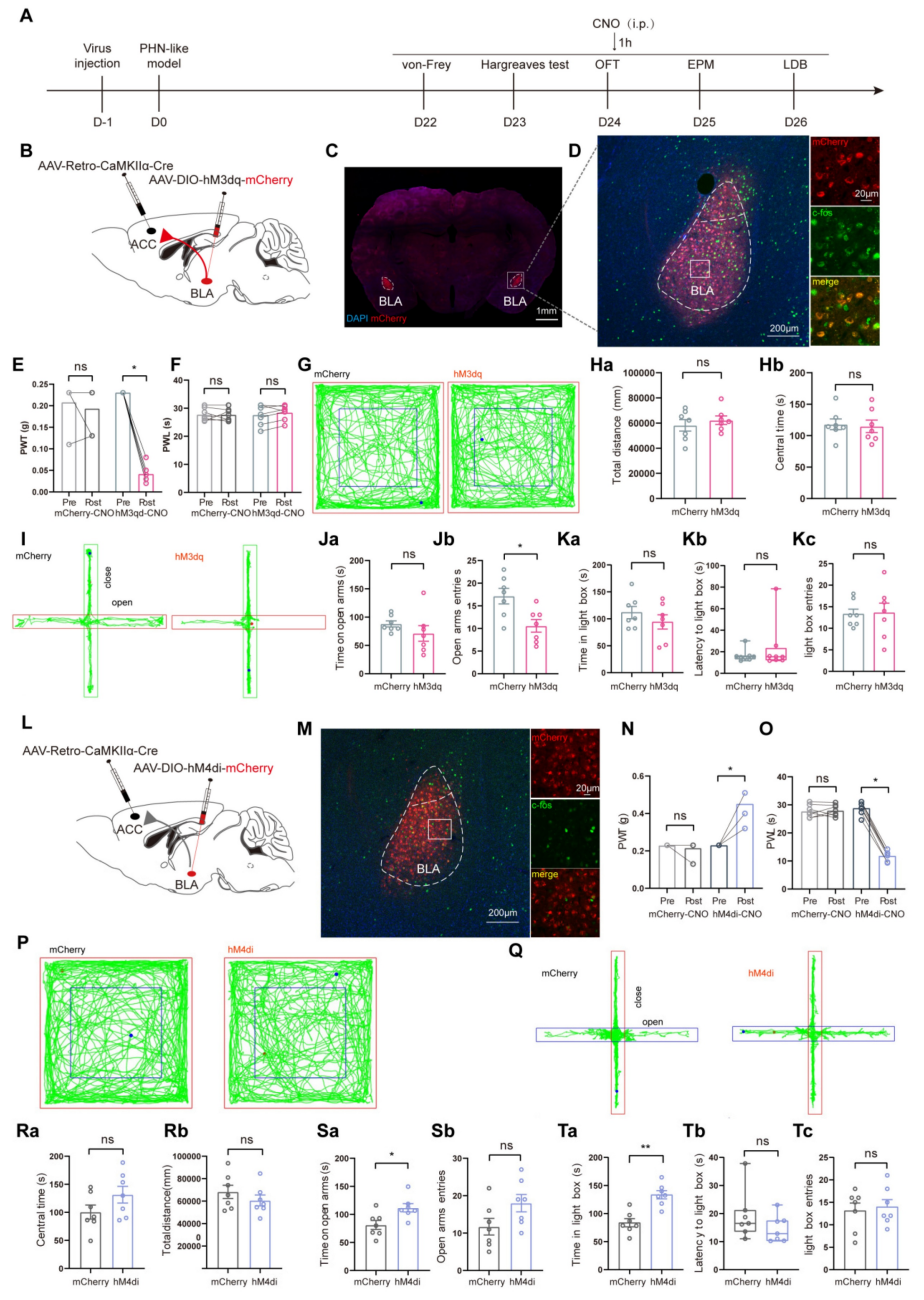
** Chemogenetic modulation of BLA^ACC^ neurons alters pain and anxiety-like behaviors in PHN comorbid anxiety-like mice.** (A) Flow diagram of chemogenetic modulation of BLA^ACC^ neurons. (B) Schematic diagram of chemogenetically activated viruses injected into BLA and ACC in WT mice. (C) Representative diagram of BLA virus expression bilaterally, scale bar 1 mm. (D) Representative diagram of neurons expressing mCherry+ in BLA co-labeled with c-fos, scale bar 200 μm. The area shown is enlarged in the white box of BLA, scale bar 20 μm. (E) stereotaxic injection of control or hM3dq virus in the PHN comorbid anxiety-like mice, and the mechanical pain thresholds (Comparing before and after CNO injection, *p* = 0.500 and *p* = 0.016 in mCherry or hM3dq-mCherry group, respectively) and (F) the thermal paw withdrawal latency (Comparing before and after CNO injection, *p* = 0.844 and *p* = 0.384 in mCherry or hM3dq-mCherry group, respectively) comparisons by intraperitoneal injection of CNO (n = 7 mice/group). (G) Representative plots of OFT trace in PHN comorbid anxiety-like mice with mCherry or hM3dq-mCherry after CNO injection. (Ha) the total distance in OFT (t = 0.732, *p* = 0.478) and (Hb) central time spent (t = 0.241, *p* = 0.814) in both groups after the injection of CNO (n = 7 mice/group). (I) Representative plots of the exploration trace in the EPM. (Ja) The duration of stay in the open arm of the EPM (t = 1.218, *p* = 0.247) and (Jb) entries into the open arm (t = 2.963, *p* = 0.012). In the LDB, (Ka) spent time in the lightbox (t = 1.007, *p* = 0.334), (Kb) latency to enter the lightbox (U = 32, *p* > 0.999), and (Kb) the entries into the lightbox (t = 0.112, *p* = 0.912) (n = 7 mice/group). (L) Schematic diagram of inhibition viruses injected into BLA and ACC in WT mice. (M) Representative diagram of neurons expressing mCherry+ in the BLA co-labeled with c-fos, scale bar 200 μm. White boxes denote magnified areas in the BLA, scale bar 20 μm. (N) Mechanical pain thresholds in PHN comorbid anxiety-like mice injected with either control or hM4di virus (Comparing before and after CNO injection, *p* > 0.999 and *p* = 0.016 in mCherry or hM4di-mCherry group, respectively) and (O) the thermal paw withdrawal latency (Comparing before and after CNO injection, *p* = 0.781 and *p* = 0.016 in mCherry or hM4di-mCherry group, respectively) (n = 7 mice/group). (P) Representative plots of OFT trace in PHN comorbid anxiety-like mice with mCherry or hM4di-mCherry after CNO injection. (Qa) Total distance in OFT (t = 1.008, *p* = 0.334) and (Qb) central time (t = 1.592, *p* = 0.137) in both groups after the injection of CNO (n = 7 mice/group). (R) Representative plots of exploration trace in the EPM in two groups after CNO injection. (Sa) Time spent in the open arm of the EPM (t = 2.795, *p* = 0.016) and (Sb) entries into the open arm (t = 1.961, *p* = 0.074) after CNO injection. (Ta) Comparison of time spent in the lightbox (t = 1.627, *p* = 0.126), (Tb) latency to enter the lightbox (U = 30, *p* = 0.878), and (Tc) entries into the lightbox (t = 1.936, *p* = 0.073) (n = 7 mice/group) in the LDB experiment. (*p < 0.05, **p < 0.01, ***p < 0.001).

Then, we examined whether activating or inhibiting BLA^ACC^ neurons could affect pain and anxiety-like behaviors in RTX mice. AAV/Retro-CaMKII-Cre viruses were injected in ACC and AAV-DIO-hM3dq-mCherry or control virus were injected into BLA bilaterally. Following intraperitoneal injections of CNO, behavioral experiments were conducted (Fig. 10A-C). IHC revealed that a majority of mCherry+ neurons were co-labeled with c-fos, demonstrating the functionality of the AAV-DIO-hM3dq-mCherry virus (Fig. 10D). In comparison to the mCherry-treated group, pain and anxiety-like behaviors were further aggravated in hM3dq-treated group after intraperitoneal injection of CNO (Fig. 10E-K). We next explored whether inhibiting the BLA^ACC^ neurons could effectively alleviate RTX-induced pain and anxiety-like behaviors. The AAV/Retro-CaMKII-Cre virus was injected into ACC, and the AAV-DIO-hM4di-mCherry or control virus was delivered bilaterally into BLA. After three weeks of viral transfection, BLA^ACC^ neurons were inhibited by CNO (Fig. 10L, M). The hM4di-treated mice showed significant rescue from mechanical and thermal pain, and their anxiety-like behaviors were rescued (Fig. 10Q, S, and T). No notable difference in total distance traveled during OFT was observed between the two groups, implying that inhibition of BLA^ACC^ neurons did not impair locomotor ability (Fig. 10P, R).

To further confirm the role of BLA^ACC^ neurons, we employed the optogenetic approach. We injected AAV/Retro-CaMKII-Cre into ACC and AAV-DIO-ChR2-mCherry in BLA, while placing optical fibers in BLA bilaterally (Fig. S3A-C). Activation of BLA^ACC^ neurons by blue laser aggravated mechanical pain in the mice (Fig. S3D). The results of OFT showed no significant differences in total distance traveled or time in the central zone between the two groups (Fig. S3F-H), suggesting no impairment in locomotor function. However, ChR2-treated mice spent less time and made fewer entries in the open arms in the EPM, indicating exacerbated anxiety-like behavior (Fig. S3I-K). We then applied an optogenetic inhibition strategy by injecting an inhibitory virus (AAV-DIO-eNPHR-mCherry). In contract to optogenetic activation, suppressing BLA^ACC^ neurons with yellow light alleviated mechanical pain in the eNPHR-treated group (Fig. S3E). In the OFT, the time in central was increased in the eNPHR-treated group, with no statistical differences identified in total distance between the two groups (Fig. S3L-N). The entries in the open arm and the time spent in the open arm increased in EPM in eNPHR-treated mice (Fig. S3O-Q). In summary, these findings provide additional evidence that the BLA^Glu^-ACC circuit was sufficient and required for the regulation of pain and anxiety-like behaviors in PHN comorbid anxiety-like mice.

## Discussion

PHN often co-occurs with psychiatric disorders such as anxiety and depression, which reinforce each other, and traditional analgesics have limited therapeutic effects on these two symptoms. Currently, clinical treatment primarily focuses on symptom management, and there is no specific pharmacological therapy available. A deeper understanding of the neurobiological mechanisms contributing to PHN-induced anxiety behavior is crucial for the development of novel therapeutic approaches. The present study indicates that the PHN-anxiety comorbidity patients exhibited reduced amygdala GM volume and diminished FC of BLA-ACC, with FC values negatively correlating with VAS and HAMA scores. Additionally, the study identified that the overactivity of BLA^Glu^ neurons was critical for maintaining mechanical pain sensitivity, thermal pain disorder, and anxiety-like behavior in PHN comorbid anxiety-like mice. Chemical and photogenetic manipulation of bilateral BLA^Glu^ neurons modulated nociceptive deficits and negative emotions in PHN comorbid anxiety-like mice. Viral tracing and electrophysiology recording further confirmed monosynaptic excitatory connections between BLA^Glu^ and ACC neurons and revealed enhanced excitatory inputs from BLA^Glu^ neurons to ACC pyramidal neurons in PHN comorbid anxiety-like mice. Further specific modulation of the BLA^Glu^-ACC circuit also led to alterations in pain and anxiety-like behaviors. These findings suggest that the structural and functional plasticity of the BLA-ACC circuit may be pivotal for the onset of PHN-related anxiety-like behaviors. Given its sufficiency and necessity in maintaining PHN-anxiety comorbidity, this circuit emerges as a potential target for clinical treatment (such as transcranial direct current stimulation, transcranial magnetic stimulation, and deep brain stimulation 37, 38) of PHN-anxiety comorbidity disorders.

There is accumulating evidence that the amygdala is a critical area involved in emotion regulation, physical responses, and pain processing 39-41. Neuroimaging studies have shown structural alterations of the amygdala in pain and affective disorders 42-45. Our study also revealed that the volumes of the amygdala were significantly reduced in PHN-anxiety patients compared with HCs. Moreover, the voxels of GM volumes in the amygdala showed the most pronounced alterations, leading us to select this brain region as an entry point. One study investigating chronic insomnia disorder patients identified distinct areas within the amygdala linked to sleeplessness and anxiety, respectively 46. Another study highlighted that different altered connectivity patterns in amygdala subregions may provide unique insights into the development of depression 47, 48. This evidence contributes to our understanding of the functional specificity of amygdala subregions. Accordingly, we divided the amygdala into three subregions based on Julich brain mapping and used these subregions as seeds to conduct a whole-brain exploration. Our results revealed a reduction in FC between left BLA and ACC, with FC values showing a negative correlation with the VAS and HAMA scores. Although several brain regions show preliminary evidence of functional lateralization in the context of pain, no area has shown greater capacity for left and right differences in input and output than the amygdala 49, 50. Numerous studies have shown that the amygdala exhibits left-right functional differences in emotion and pain processing: the left amygdala is generally involved in fine-grained emotional encoding (language-related emotional processing) and negative emotion regulation 51-54, which may explain why structural and functional changes in left BLA correlate with pain intensity (VAS) and anxiety (HAMA) scores. In contrast, the right amygdala is more involved in non-verbal emotional responses (facial expression recognition) and autonomic regulation 55, with structural changes likely reflecting broader emotional states rather than specific pain-anxiety comorbidity. Furthermore, our previous research has shown that PHN had an effective connection change from the amygdala subregion to the ACC and putamen compared to HCs by the Granger causality analysis 56. Consequently, we concentrated our study on the BLA-ACC circuit. ACC involvement in pain perception and anxiety 18. Previous fMRI studies have shown reduced GM volumes and altered FC in ACC in patients with pain and anxiety 57-59, while other clinical research has suggested that ACC also participates in processing emotions associated with neuropathic pain 60, 61. Thus, the reduction of FC between BLA and ACC in PHN-anxiety comorbidity patients may be attributed to the combined effects of pain and anxiety, enhancing our understanding of the role of this circuit in PHN-related affective disorders.

Previous studies have shown that systemic treatment of RTX could produce mechanical and thermal disturbance in mice or rats that resemble the unique clinical features of PHN rather than mimicking its specific etiological mechanisms 30, 62. In this study, the RTX model was used to mimic the pain and anxiety behavioral phenotypes associated with PHN. Indeed, pain is a complex, multidimensional experience that encompasses emotional components, and PHN is frequently accompanied by emotional problems such as anxiety, underscoring the importance of investigating the central mechanisms driving the pathogenesis of PHN. In this study, we used Fos-CreER::Ai9 mice to label neurons associated with PHN comorbid anxiety-like behaviors. Based on the results of fMRI VBM analysis, we explored whether brain regions exhibiting volume changes in PHN-anxiety patients also displayed c-fos protein differences in the mouse model. Our findings revealed a significant elevation in the number of c-fos positive neurons in BLA, ACC, and mPFC in PHN comorbid anxiety-like mice compared to the sham group. Besides, we found the overactivity of BLA^Glu^ neurons in PHN comorbid anxiety-like mice and confirmed that modulating BLA^Glu^ neuron activity by opto/chemogenetic techniques could alter pain and anxiety-like behaviors. Previous studies have highlighted the critical role of BLA in pain and emotional processing 15, 63, with optical fiber photometry investigations demonstrating that activation of BLA neural ensemble was necessary for the negative affective valence of pain 64, while lesions in BLA could prevent the development of pain chronicity in neuropathically painful rats 65. Additionally, cannulation and administration of the NMDA receptor antagonist (MK-801) delivery in BLA produced analgesic effects, whereas transitory pain was induced by infusion of the GABA(A) receptor antagonist bicuculline 66. These studies align with our findings that BLA neurons are involved in pain and anxiety. However, we found that activation of BLA neurons in PHN comorbid anxiety-like mice exacerbated pain but did not significantly affect anxiety-like behaviors (Fig. 5 and Fig. S3). We attribute this to a ceiling effect, as immunohistochemistry and *in vitro* electrophysiology recordings demonstrated that BLA^Glu^ neurons were already activated in RTX-treated mice. Further activation of these neurons in RTX-treated mice was less likely to induce behavioral changes. For example, Corder *et al.* 64 found that baseline activity of amygdala neurons was elevated in neuropathic pain mice, making further stimulation less likely to induce behavioral changes. Additionally, the anxiety-related functions of the BLA may depend on its interactions with other brain regions (central amygdala, mPFC, or hypothalamus) 67, 68. In the PHN-anxiety comorbidity state, these downstream regions may counteract the effects of BLA activation through adaptive mechanisms (synaptic plasticity or changes in neurotransmitter release) 69, 70. Overall, our findings support the essential role of BLA^Glu^ neurons in modulating PHN comorbid anxiety-like behaviors.

Our clinical studies showed that the FC of BLA-ACC was reduced in PHN-anxiety patients. Further, we revealed that the BLA^Glu^ neurons formed excitatory monosynaptic connections with ACC, as demonstrated by viral tracing and *in vitro* electrophysiology recordings. Compared to the sham group, the PHN comorbid anxiety-like mice exhibited higher excitability of ACC pyramidal neurons. It has been well-established that the BLA is anatomically connected to brain regions involved in pain and anxiety 70, and increased excitatory synaptic transmission in BLA plays a crucial role in pain processing 71. These findings suggest that ACC might serve as a vital downstream brain area, integral to the proper functioning of BLA in PHN-anxiety comorbidity. The ACC has been recognized as a hub for pain and emotion processing 18, 72, as neurons in ACC displayed notable increases in firing rate and burst activity in neuropathic pain with anxiety and depression 73. In addition, inhibition of ACC pyramidal neurons was sufficient to alleviate neuropathic pain-induced aversive and anxiety-like behaviors 73, 74. In this study, we found an increase in the amplitude of oEPSC as well as a decrease in PPR in ACC II/III layers pyramidal neurons in PHN comorbid anxiety-like mice compared to the sham group, indicating enhanced presynaptic transmitter release. Furthermore, optogenetic/chemogenetic activation/inhibition of the terminal of BLA^Glu^ neuron in the ACC exacerbated/alleviated pain and anxiety-like behaviors in PHN comorbid anxiety-like mice. Our results are consistent with prior reports showing presynaptic adaptations in the excitatory transmission of ACC II/III layers pyramidal neurons in pain conditions 36, 75.

We then investigated the role of the BLA^ACC^ neurons in the development of anxiety-like behaviors induced by RTX. Activation of BLA^ACC^ neurons in PHN comorbid anxiety-like mice with optogenetic/chemogenetic techniques further exacerbated both pain and anxiety-like behaviors. In contrast, activating BLA^Glu^ neurons specifically worsened pain behavior, while no significant effect was observed on anxiety behavior. These results suggest that ACC-projecting BLA neurons may selectively regulate PHN-anxiety behaviors. Inhibition of the BLA^ACC^ neurons, on the other hand, alleviated pain and anxiety behaviors in PHN comorbid anxiety-like mice, implying that the excitatory changes in the BLA^ACC^ neuron are driven by the combined effects of pain and anxiety. Becker *et al.* 25 showed that optogenetic inhibition of the BLA-ACC circuit alleviated depression-like behaviors induced by chronic pain, whereas long-term activation of this circuit in naive mice led to depression-like behaviors. Valentinova K's study 36 concluded that the BLA-ACC circuit was involved in the cognitive aspect of pain. Our results are consistent with these findings, supporting the idea that the BLA^Glu^-ACC circuit may play a role in both pain hypersensitivity and emotional disorder under the PHN condition.

In conclusion, our study demonstrated that both the structure and function of the amygdala were altered in PHN-anxiety patients. In mice, PHN-anxiety comorbidity resulted in overactivity of BLA and ACC neurons. Inhibition of the BLA^Glu^-ACC circuit alleviated pain and anxiety behaviors related to PHN. These findings imply that the BLA^Glu^-ACC circuit may represent a potential therapeutic target in the management of PHN.

## Supplementary Material

Supplementary figures and tables.
