# Supplementary material for: The basolateral amygdala-anterior cingulate cortex circuit contributes to postherpetic neuralgia-anxiety comorbidity
Source: Theranostics. 2025 Mar 21;15(10):4614–37. doi: 10.7150/thno.111130 (PMC11984391; doi:10.7150/thno.111130)
Supplement: Supplementary file 1 — Supplementary figures and tables. [file thnov15p4614s1.pdf]

Supplementary Table 1 Demographic data and clinical scores of PHN-anxiety patients and HC

Clinical information	HC	PHN-anxiety	Statistic results	*P*-value
	n = 40	n = 41		
Age (years)	66.20 ± 6.79	64.88 ± 10.32	t = 0.679	0.499
Sex (M/F)	19/21	22/19	c^2 = 0.307	0.579
Education(years)	9(6, 9)	9(6, 9)	U = 698	0.197
Illness duration (days)	NA	60(30, 150)		
VAS score	NA	6 (6, 6.5)		
HAMA score	5.92 ± 1.06	30.46 ± 8.68	t = 17.77	＜0.0001

VAS: Visual Analog Scale; HAMA: Hamilton Anxiety Scale.

Data satisfying normal distribution were described as mean ± SD, otherwise denoted as median (interquartile spacing). t, U, and c^2 denote unpaired t test, Mann-Whitney U test, and chi-square test statistics, respectively.

Supplementary Table 2. Reductions of GM volume in PHN-anxiety patients compared to HCs (whole-brain VBM analysis)

Region AAL (BA)	Hemisphere	MNI coordinates (mm)			Peak intensity	Cluster size (voxels)	*P*
		x	y	z			
Amygdala/ Hippocampus	R	33	-6	-18	8.25	1678	0.000
Amygdala/ Hippocampus	L	-24	-13.5	-13.5	7.90	1090	0.000
Frontal_Med_Orb	L	-4.5	37.5	-12	8.64	1928	0.000
Fusiform	R	18	-33	-1.5	8.19	738	0.000
Fusiform	L	-22.5	-48	-12	6.91	170	0.000
Lingual	L	-18	-69	-13.5	7.63	276	0.000
Thalamus	L	0	-3	-3	7.06	370	0.000
Cingulum_Mid	L	0	19.5	42	7.23	236	0.000
Insula	R	37.5	24	0	7.15	100	0.000
Postcentral	L	-52.5	-15	34.5	7.28	114	0.000

Supplementary Table 3: Brain areas with reduced FC in PHN-anxiety patients compared to HC

Regions of interest	Brain region		Cluster size (number of voxels)	Peak intensity	MNI coordinates		
					X	Y	Z
Basolateral amygdala (L)	Anterior Cingulate/ Inferior Frontal Gyrus	R	439	-4.05	-6	-3	-12

Basolateral amygdala (R)	Temporal Lobe	L	211	-4.72	-57	-12	-6
Centromedian amygdala (L)	Thalamus	L	187	-4.74	9	18	15
Centromedian amygdala (R)	Frontal Lobe	L/R	92	-4.44	3	36	-18

Results were corrected for thresholds using Gaussian random field theory (GRF) at the two-tailed, voxel-level $P <$ 0.001 and cluster level $P < 0.05$.

Abbreviations: R, right hemisphere; L, left hemisphere.

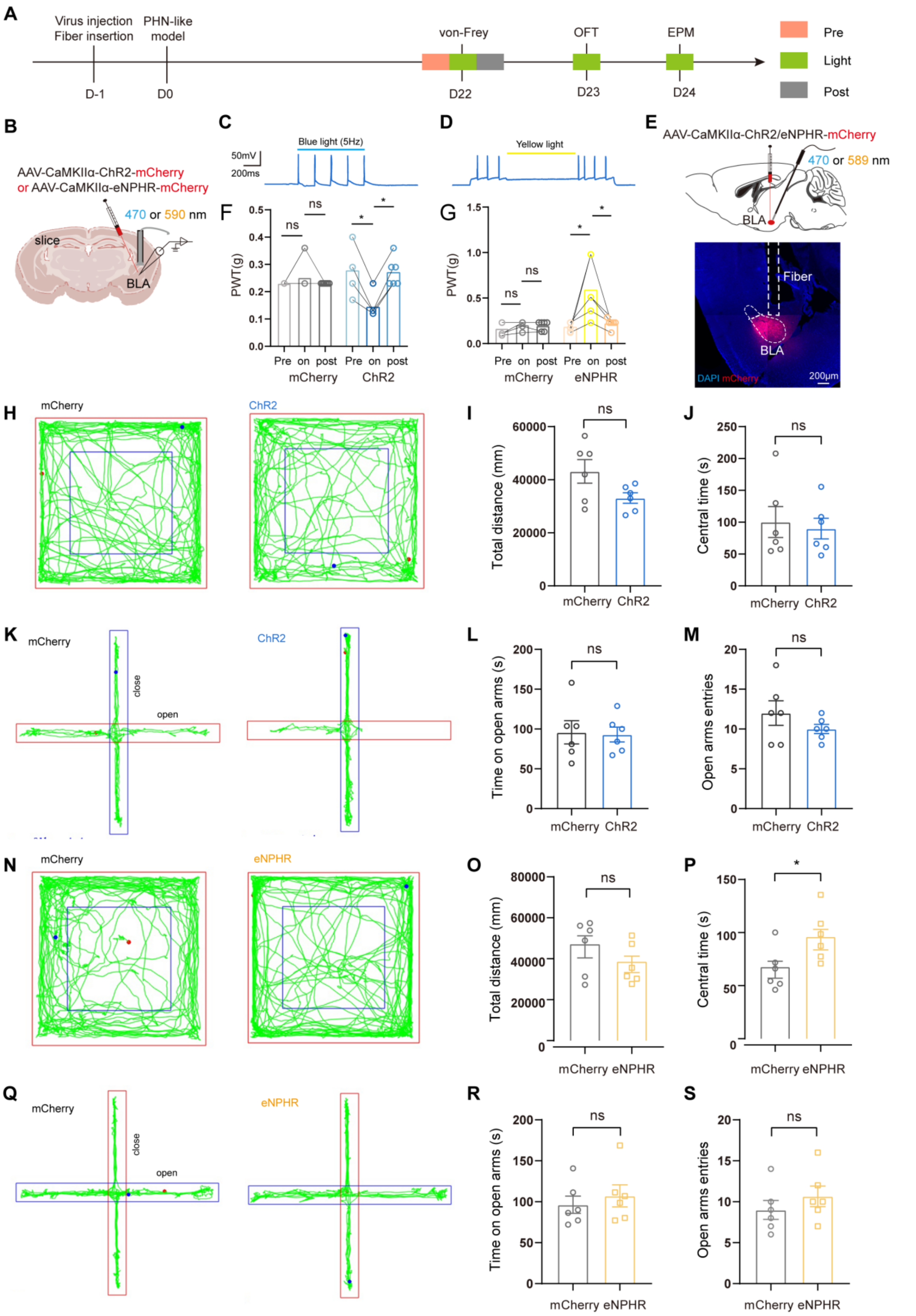

Supplementary Fig. 1 Optogenetic modulation of BLA^{Glu} neurons alters pain and anxiety-like behaviors in PHN comorbid anxiety-like mice. (A) Schedule of experiments. (B) Schematic diagram of virus injection and electrophysiology. (C) Action potentials generated by 5 Hz blue laser-induced ChR2-expressing neurons in BLA.

(D) the hyperpolarized membrane potentials induced by yellow laser in BLA neurons infected with eNpHR-mCherry in brain slices. (E) Schematic of virus injection (top) and representative image of virus expression (bottom), scale bar 200 μm. (F) Mechanical pain thresholds in PHN-anxiety mice injected with either control or ChR2 virus after blue light (control pre vs on $p > 0.999$, $p > 0.999$ for on vs post; experimental pre vs on $p = 0.031$, on vs post $p = 0.031$) (n = 6 mice/group). (G) Mechanical pain thresholds after yellow light stimulation with the injection of control or eNPHR virus in PHN-anxiety mice (controls: pre vs on $p = 0.250$, on vs post $p > 0.999$; eNPHR groups: pre vs on $p = 0.031$, on vs post $p = 0.031$) (n = 6 mice/group). (H) Representative plots of OFT trace after light on in PHN-anxiety mice with mCherry or ChR2-mCherry. (I) OFT total distance (t = 2.055, $p = 0.067$) and (J) time in the central region (t = 0.356, $p = 0.729$) for both groups of mice (n = 6 mice/group). (K) Representative plots of exploring trace in the EPM after transfection with mCherry or ChR2-mCherry during light on. Time spent in the open arm of the EPM (L) (t = 0.156, $p = 0.879$) and number of entries into the open arm (M) (t = 1.210, $p = 0.254$) of both groups (n = 6 mice/group). (N) Representative plots of OFT trace after light on in PHN-anxiety mice transfected with mCherry or eNPHR-mCherry. Total OFT (O) travel (t = 1.274, $p = 0.231$) and (P) central region dwell time (t = 2.269 $p = 0.046$) for both groups of mice (n = 6 mice/group). (Q) Representative maps of PHN-anxiety mice exploring trace in the EPM after transfection with mCherry or eNPHR-mCherry given light. Time spent in the open arm of the EPM (R) (t = 0.635, $p = 0.539$) and number of entries into the open arm (S) (t = 0.977, $p = 0.352$) for both groups of mice (n = 6 mice/group). (*$p < 0.05$, **$p < 0.01$, ***$p < 0.001$).

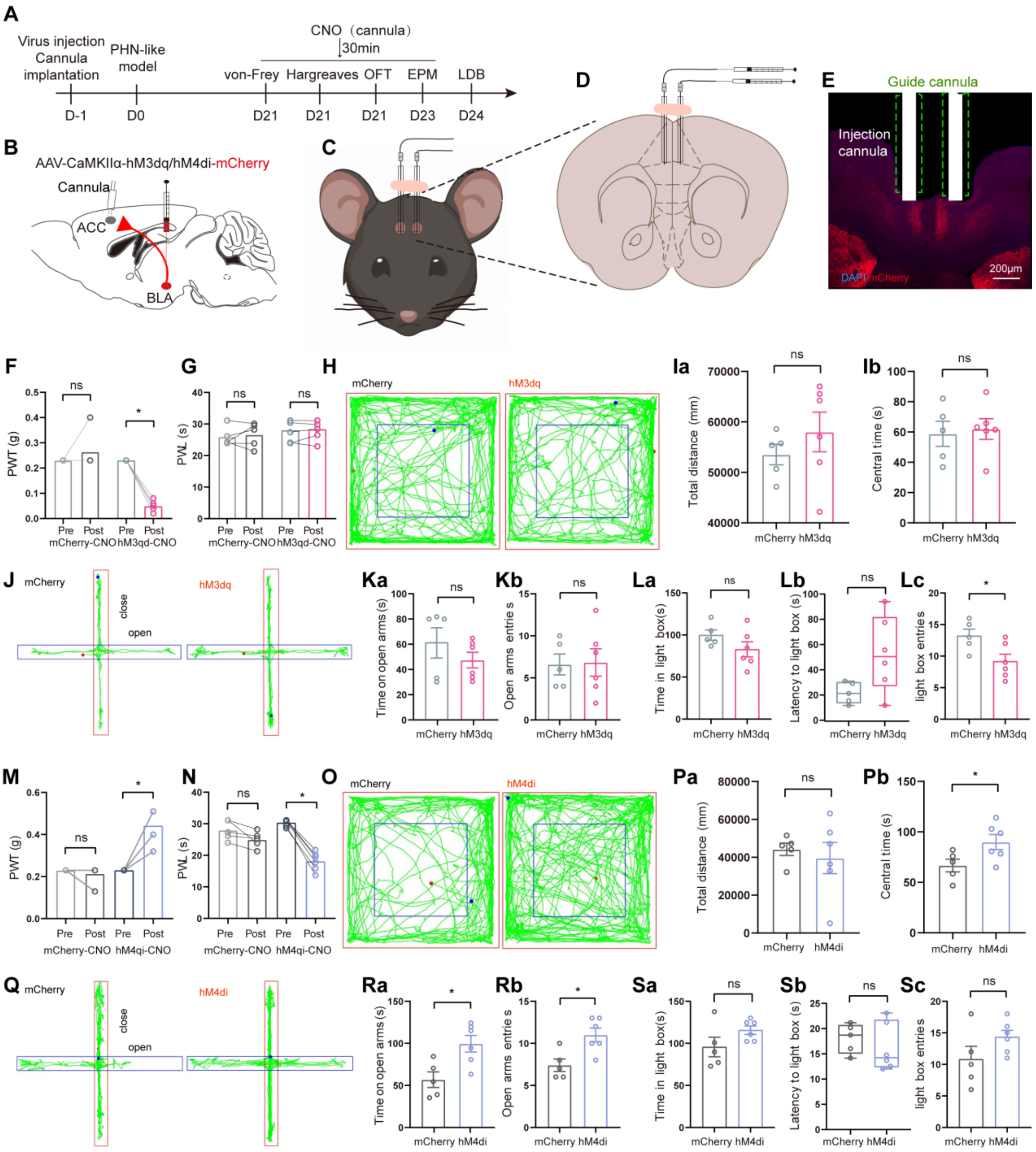

Supplementary Fig. 2 Chemogenetic modulation of BLA^{Glu}-ACC projections alters pain and anxiety-like behaviors in PHN comorbid anxiety-like mice. (A) Timeline of chemogenetic modulation of BLA^{Glu}-ACC projections. (B-D) Schematic illustration of chemogenetic of the BLA^{Glu}-ACC projections by viral injection and cannulation. (E) Representative coronal brain sections showing axon terminals labeled by chemogenetic viruses in the ACC and the location of cannulation, scale bar 500 μm. (F) Comparison of mechanical pain (mCherry group: $p > 0.999$; hM3dq group: $p = 0.031$) and (G) the latency of thermal pain (mCherry group: $p > 0.999$; hM3dq group: $p = 0.875$) between the mCherry and the hM3dq-mCherry group before and after cannula injection of CNO. (H) Representative plots of OFT trace in two groups of mice after microinjection of CNO. In the OFT, (Ia) the total distance (t = 0.951, $p = 0.366$) and (Ib) time in the center region (t = 0.293, $p = 0.776$) of both groups after CNO injection. (J) Representative plots of trace in the EPM for both groups of mice. In the EPM, (Ka) the duration of stay

in the open arm (t = 1.110, $p = 0.296$) and (Kb) entries into the open arm (t = 0.110, $p = 0.915$) in both groups after CNO injection. In the LDB experiment, (La) the time spent in the lightbox (t = 1.485, $p = 0.172$), (Lb) latency to enter the lightbox (U = 4, $p = 0.052$), and (Lc) entries into the lightbox (t = 2.545, $p = 0.031$) in both groups (n = 5 mice in the mCherry group, and n = 6 mice in the hM4di-mCherry group). Comparison of (M) mechanical pain (mCherry group: $p > 0.999$; hM4di group: $p = 0.031$) and (N) the latency of thermal pain (mCherry group: $p = 0.062$; hM4di group: $p = 0.031$) in the mCherry and the hM4di-mCherry group before and after injection of CNO. (O) Representative plots of OFT trace after microinjection of CNO in the mCherry or hM4di-mCherry groups. In the OFT, (Pa) the total distance (t = 0.484, $p = 0.640$) and (Pb) time in the center region (t = 3.002, $p = 0.015$) of both groups after CNO injection. (Q) Representative plots of trace in the EPM for both groups of mice. In the EPM, (Ra) the duration in the open arm (t = 3.105, $p = 0.013$) and (Rb) entries into the open arm (t = 3.193, $p = 0.011$) in both groups after the injection of CNO. In the LDB experiment, (Sa) the time spent in the lightbox (t = 1.696, $p = 0.124$), (Sb) latency to enter the lightbox (U = 11, $p = 0.537$), and (Sc) entries into the lightbox (t = 2.361, $p = 0.039$) in both groups (n = 5 mice in the mCherry group, and n = 6 mice in the hM4di-mCherry group). (*$p < 0.05$, **$p < 0.01$, ***$p < 0.001$).

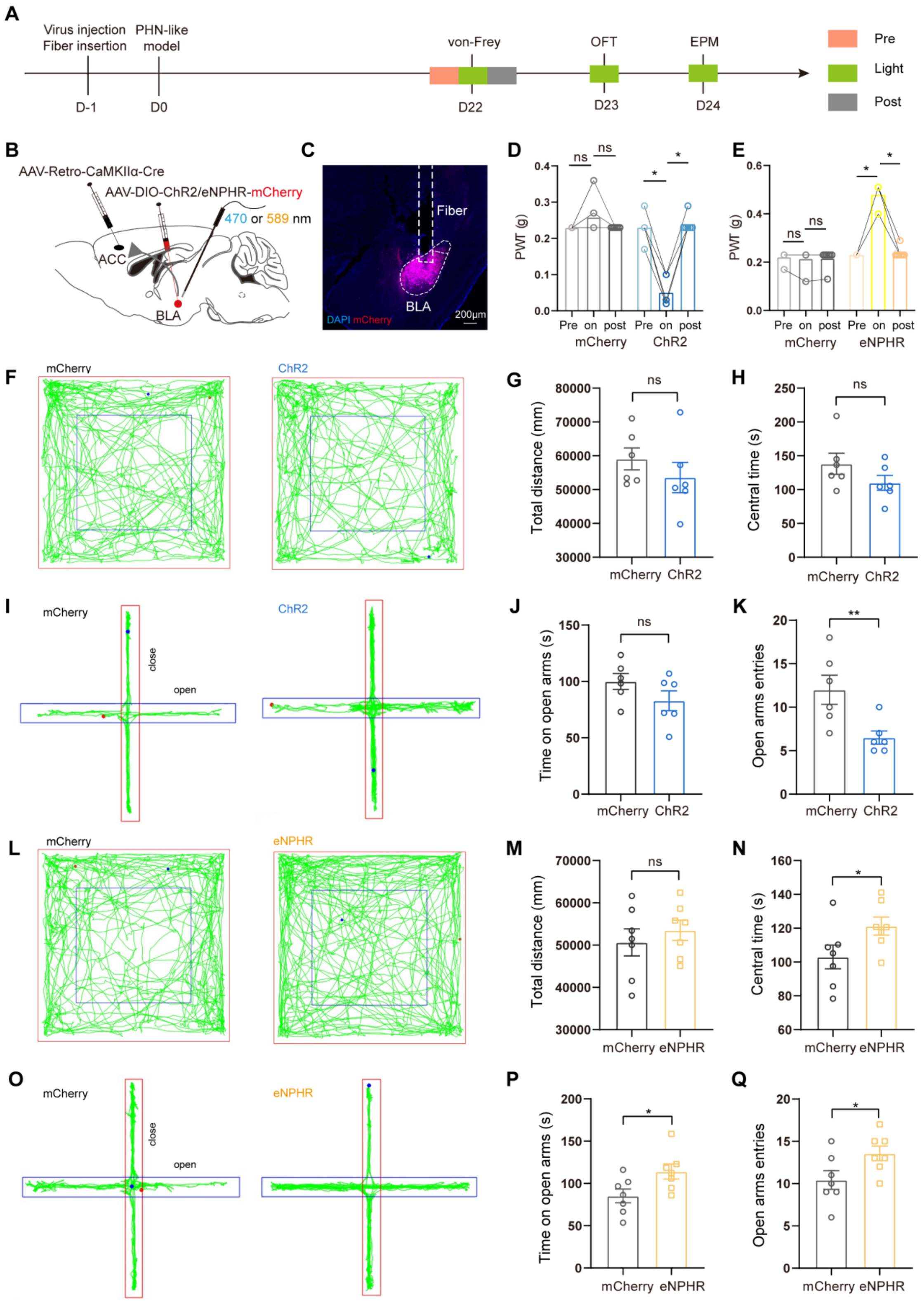

Supplementary Fig. 3 Optogenetic modulation of BLA^{ACC} alters pain and anxiety-like behaviors in PHN comorbid anxiety-like mice. (A) Flowchart of the experiment of optogenetic modulation of BLA^{ACC}. (B) Schematic diagram of virus injection and fiber placement. (C) Schematic diagram of BLA virus expression and fiber optic

placement, scale bar 200 μm. (D) Mechanical pain thresholds in PHN comorbid anxiety-like mice after injection of control or ChR2 viruses activated by blue light (in the control group: pre vs on $p = 0.242$, on vs post $p = 0.500$; in experimental group: pre vs on $p = 0.031$, on vs post $p = 0.031$) (n = 6 mice/group). (E) Mechanical pain thresholds after yellow light stimulation (in the control group: pre vs on $p = 0.356$, on vs post $p = 0.356$; in the experimental group: pre vs on $p = 0.016$, on vs post $p = 0.015$) (n = 7 mice/group). (F) Representative plots of OFT trace in PHN comorbid anxiety-like mice with mCherry or ChR2-mCherry. (G) Total distance in OFT (t = 0.994, $p = 0.344$) and (H) central time (t = 1.473, $p = 0.172$) for both groups (n = 6 mice/group). (I) Representative maps of PHN comorbid anxiety-like mice with mCherry or ChR2-mCherry exploring trace in the EPM. (J) In the EPM, time spent in the open arm (t = 1.52, $p = 0.161$) and (K) entries into the open arm (t = 2.990, $p = 0.014$) for both groups (n = 6 mice/group). (L) Representative plots of OFT trace in PHN comorbid anxiety-like mice with mCherry or eNPHR-mCherry. (M) Total distance in OFT (t = 0.717, $p = 0.487$) and (N) central time (t = 2.278 $p = 0.038$) for both groups (n = 6 mice/group). (Q) Representative exploring trace in the EPM in PHN comorbid anxiety-like mice with mCherry or eNPHR-mCherry. (P) Time spent in the open arm of (t = 2.393, $p = 0.034$) and (Q) entries into the open arm (t = 2.230, $p = 0.045$) in EPM of both groups (n = 7 mice/group). (*$p < 0.05$, **$p < 0.01$, ***$p < 0.001$).
